# Homogeneous Nanoparticles of Multimetallic Phosphides via Precursor Tuning: Ternary and Quaternary M_2_P Phases (M = Fe, Co, Ni)

**DOI:** 10.1021/acsnanoscienceau.2c00025

**Published:** 2022-08-09

**Authors:** Tepora Su’a, Mikaylah N. Poli, Stephanie L. Brock

**Affiliations:** Department of Chemistry, Wayne State University, Detroit, Michigan 48202, United States

**Keywords:** synthesis, phosphidation rate, trimetallic phosphide, solid solution, composition control

## Abstract

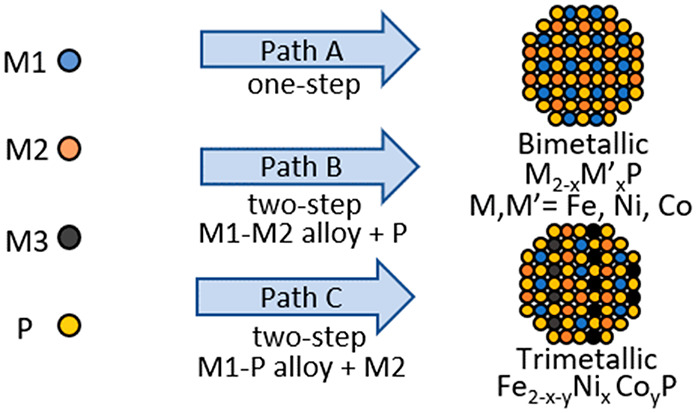

Transition metal phosphides (TMPs) are a highly investigated class of nanomaterials due to their unique magnetic and catalytic properties. Although robust and reproducible synthetic routes to narrow polydispersity monometallic phosphide nanoparticles (M_2_P; M = Fe, Co, Ni) have been established, the preparation of multimetallic nanoparticle phases (M_2–*x*_M′_*x*_P; M, M′ = Fe, Co, Ni) remains a significant challenge. Colloidal syntheses employ zero-valent metal carbonyl or multivalent acetylacetonate salt precursors in combination with trioctylphosphine as the source of phosphorus, oleylamine as the reducing agent, and additional solvents such as octadecene or octyl ether as “noncoordinating” cosolvents. Understanding how these different metal precursors behave in identical reaction environments is critical to assessing the role the relative reactivity of the metal precursor plays in synthesizing complex, homogeneous multimetallic TMP phases. In this study, phosphorus incorporation as a function of temperature and time was evaluated to probe how the relative rate of phosphidation of organometallic carbonyl and acetylacetonate salt precursors influences the homogeneous formation of bimetallic phosphide phases (M_2–*x*_M′_*x*_P; M, M′ = Fe, Co, Ni). From the relative rate of phosphidation studies, we found that where reactivity with TOP for the various metal precursors differs significantly, prealloying steps are necessary to isolate the desired bimetallic phosphide phase. These insights were then translated to establish streamlined synthetic protocols for the formation of new trimetallic Fe_2–*x*–*y*_Ni_*x*_Co_*y*_P phases.

## Introduction

As a class, transition metal pnictides (pnicogen = P, As, Sb, or Bi) are the subject of widespread research in diverse fields including magnetocalorics, catalysis, and thermoelectrics, owing to extensive flexibility in the compositions and structures that can be adopted. Thus, MnAs is a promising material for near-room-temperature magnetic refrigeration;^1,2^ Ni_2_P is an excellent hydrodesulfurization catalyst,^3^ and CoSb_3_ is an archetypal thermoelectric.^4^ The properties, both intrinsic and extrinsic, can be further refined by reducing the size of the solid to the nanoscale regime.^5−7^ Understanding how size manipulation on this length-scale affects the relevant ensemble properties, be they magnetic, catalytic, electronic, etc., is predicated on access to size-controlled, narrow polydispersity samples. Accordingly, there is a need for synthetic methods to transition metal pnictides that enable this kind of exquisite control. To date, considerable progress has been achieved in binary transition metal phosphides of the 3d metals, including Mn, Fe, Co, and Ni. Indeed, the synthesis of monometallic M_2_P-type systems (M = Fe, Co, Ni) has been extensively investigated,^8−13^ motivated in large part by their activity for electrocatalytic water splitting, which approaches, and in some cases surpasses, that of the traditional noble metal catalysts.

The interest in phosphides as catalysts has triggered a surge of investigations of multimetallic phosphides, seeking to achieve synergy between two or more metals and enable more efficient and/or more stable catalysts. However, synthetic pathways that can compensate for differing metal reactivity to ensure homogeneous solid solutions are lacking, particularly when more than two metals are in play. Indeed, many of the phosphides prepared for catalytic studies demonstrate compositional inhomogeneity and polydispersity, particularly when synthesized directly on supports.^14−19^ Thus, streamlining the synthesis of single-phase, discrete, multimetallic transition metal phosphide nanoparticle phases remains a significant challenge.

Despite the synthetic knowledge gap, substitution of additional elemental components into monometallic TMP systems, where successful, has often shown increased tunability and enhanced performance of the materials’ properties. For example, Ni_2–*x*_Co_*x*_P nanoparticles outperform the monometallic Ni_2_P and Co_2_P phases for water oxidation catalysis.^20^ In addition, bimetallic Fe_2–*x*_Ni_*x*_P and Ni_2–*x*_Co_*x*_P phases have shown excellent stability and enhanced activity as water reduction catalysts.^21−23^ There have also been a few reports of multimetallic Fe–Co–Ni phosphides displaying improved activity toward water oxidation; however, inhomogeneous phase segregation and polydispersity make it difficult to rationalize the source of the enhanced catalytic properties.^14,15^ Recently, the Zhang group developed a general strategy to synthesize discrete bimetallic CoMP_*x*_ (M = Fe, Ni, Mn, Cu) nanomaterials using a two-step conversion process. However, they only reported the formation of one trimetallic composition, Co_1.01_Fe_0.43_Ni_0.56_P_*x*_.^24^

As a means to streamline the formation of complex TMP phases, we aim to understand the synthetic pathways enabling the formation of single-phase monometallic transition metal phosphides of Fe, Co, and Ni and use this knowledge to devise targeted pathways to bimetallic and trimetallic phosphides. The homogeneity, composition, and size of TMP nanoparticles synthesized utilizing colloidal synthesis techniques where multiple components are present are dependent on many factors including (but not limited to) the relative reactivity of the individual components with each other, reaction temperature, and reaction time. Typical colloidal synthesis procedures for Fe_2_P, Co_2_P, and Ni_2_P nanomaterials employ zero-valent metal carbonyl or cyclooctadiene (COD) (Fe(CO)_5_,^8,25,26^ Co_2_(CO)_8_,^10,27^ and Ni(COD)_2_^28^) or multivalent acetylacetonate salt precursors (Fe(acac)_3_,^29^ Co(acac)_2_,^11^ and Ni(acac)_2_^30−32^) in combination with trioctylphosphine as the source of phosphorus, most commonly oleylamine as the reducing agent/surfactant, and additional solvents such as octadecene or octyl ether as “noncoordinating” cosolvents. Understanding how these different metal precursors behave in identical reaction environments is critical to assessing the role the relative reactivity of the metal precursor plays in synthesizing compositionally homogeneous multimetallic TMP phases.

**Scheme 1:**
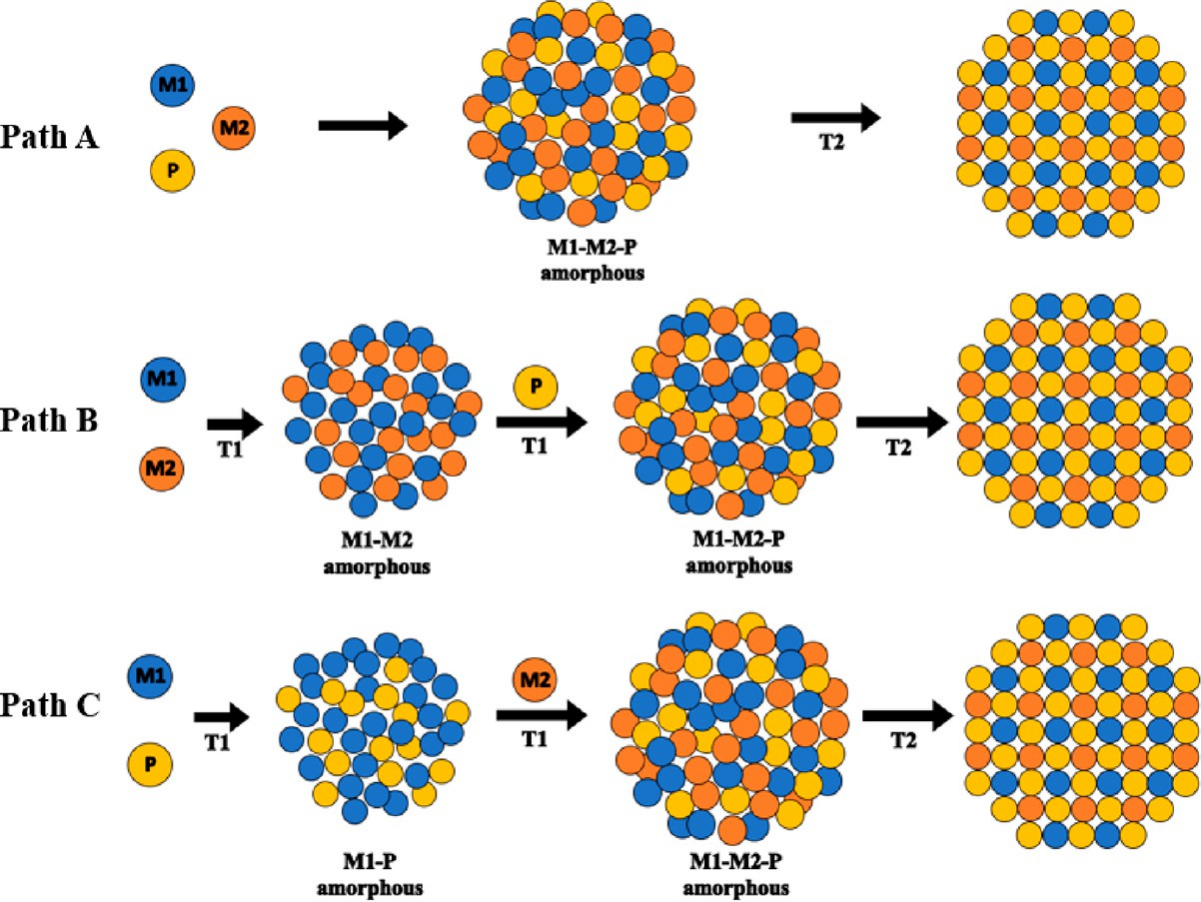
Potential Reaction Pathways Leading to the Formation of Bimetallic Transition Metal Phosphide Systems Path A: One-step reaction where both M1 and M2 are in combination with TOP (P-source) at the outset of the reaction. Path B: Two-step reaction where M1 and M2 are alloyed at the outset of the reaction to form an amorphous phase and TOP is added in an additional step. Path C: Two-step reaction where M1 is alloyed with TOP to produce an M1-P amorphous alloy and M2 is added in an additional step.

In this work, we present a comprehensive evaluation of how the relative reactivity of the individual metal precursors involved in synthesizing monometallic TMP systems (M_2_P = Fe, Co, Ni) affects the successful formation of the desired bimetallic TMP phase (Fe_2–*x*_Ni_*x*_P, Co_2–*x*_Fe_*x*_P, and Ni_2–*x*_Co_*x*_P). We show that the relative phosphidation rate of the individual metal precursors is a good guide for deciding whether single-step reactions can be achieved or whether a two-step process involving a prealloying step is needed. In this context, we consider three different approaches, as shown in Scheme 1, a one-step “heat-up” synthesis in which all reagents are introduced at the outset (Path A), a two-step process in which metal alloys are produced by “heat-up” synthesis of metal precursors followed by high temperature phosphidation (Path B), and a two-step reaction in which a single metal is phosphided by “heat-up” synthesis, followed by injection of the second metal (Path C). Notably, we find that when phosphidation rates are comparable, as for Ni and Co in Ni_2–*x*_Co_*x*_P, Path A leads to homogeneous particles, but when they are not comparable, as is the case for Fe relative to Ni or Co, Path B or C is needed. Finally, we employed the insights from the studies of bimetallic systems to target new trimetallic TMP phases of Fe_2–*x*–*y*_Ni_*x*_Co_*y*_P.

## Experimental Section

### Materials

Nickel(II) acetylacetonate (Ni(acac)_2_, 95%, Sigma-Aldrich), cobalt(II) acetylacetonate (Co(acac)_2_, 99%, Sigma-Aldrich), iron(III) acetylacetonate (Fe(acac)_3_, 97%, Sigma-Aldrich), iron(0) pentacarbonyl (Fe(CO)_5_, 99.99%, Sigma-Aldrich), dicobalt octylcarbonyl (Co_2_(CO)_8_, >90%, Sigma-Aldrich), tri-n-octylphosphine (TOP, (P(oct)_3_, 97% STREM), oleylamine (OAm, C_18_H_37_N, 70%, Sigma-Aldrich), octylether (C_16_H_34_O, 90%, TCI America), 1-octadecene (ODE, C_18_H_36_, 90% tech, Sigma-Aldrich), chloroform (CHCl_3_, 99%, Fisher Scientific), ethanol (C_2_H_5_OH, 200 proof, Decon Laboratories), and hexane (C_6_H_14_, >98.5%, Fisher Scientific) were used. Fe(CO)_5_, Co_2_(CO)_8_, TOP, and ODE were stored in a glovebox under argon. Oleylamine was further dried over molecular sieves, while all other chemicals were used as received.

### Synthesis of Monometallic Phosphides (M_2_P; M = Fe, Ni, Co)

All reactions were carried out using Schlenk line techniques under argon. In a typical one-pot reaction to synthesize monometallic phosphides, 2 mmol of the respective metal precursor was added to 2 mL of dried OAm, 3 mL of ODE, and 5 mL of TOP in a 100 mL two-neck Schlenk flask, coupled with a 12 in. condenser. The flask was connected to a Schlenk line within a fume hood and placed on a heating mantle regulated by a temperature controller. An internal thermocouple was employed, and the flask was insulated using glass wool. Under constant stirring, the reaction flask was degassed at 110 °C for 20 min and then purged with Ar at 110 °C for 20 min. To synthesize the intermediate, directly after the degas/purge step, the temperature was increased to 230 °C for 1 h. To synthesize the final product, directly after the degas/purge step, the temperature was increased to 300 °C for 1 h. The intermediates and final products were allowed to cool naturally to room temperature and isolated by precipitation with ethanol. The precipitate was then redispersed in hexane or chloroform with 5–10 min sonication followed by reprecipitation with ethanol. Reprecipitated particles were isolated via centrifugation, and the entire dispersion/precipitation process was repeated three times.

In a typical two-step synthesis, 2 mmol of the respective precursor was combined with 2 mL of OAm and 5 mL of ODE in a two-neck Schlenk flask. Under constant stirring, the reaction mixture was degassed at 110 °C for 20 min and purged with Ar at 110 °C for 20 min. The temperature was increased to 230 °C and maintained for 1 h, and then TOP was injected and the mixture was allowed to age for 30 min. After 30 min, the temperature was increased to 300 °C and kept for 1 h. Resultant particles were allowed to cool naturally and isolated as described for the one-pot reactions.

### Phosphidation Rate Aliquot Studies

A total of 2 mmol of the respective metal precursor was added to 2 mL of dried OAm, 3 mL of ODE, and 5 mL of TOP (10.7 mmol) in a 100 mL two-neck Schlenk flask, coupled with a 12 in. condenser. Under constant stirring, the reaction mixture was degassed at 110 °C for 20 min and purged with Ar at 110 °C for 20 min. The temperature was then increased to 230 °C, and 0.2 mL was retrieved from the reaction mixture and injected into 3 mL of cold hexane. Aliquots were subsequently retrieved in a similar fashion at 285, 295, 300 (0 min), 300 (5 min), 300 (10 min), 300 (15 min), and 300 °C (30 min). A slow ramp rate of 5 °C/10 min was used to prevent temperature overshoot. Each aliquot was taken immediately after the solution reached the target temperature. Then after 1 min at each of the targeted reaction temperatures (the 1 min was taken into account to allow for temperature stabilization), the temperature was increased to the next target. For the 300 °C step, the same ramp rate was used, with aliquots taken immediately after the temperature reached 300 °C (0 min), 5 min after reaching 300 °C (5 min), etc. Resultant particles were isolated as described for the one-pot reactions.

### Synthesis of Bimetallic Phosphides (M_2–*x*_M′_*x*_P; M, M′ = Fe, Ni, Co)

Bimetallic phosphides were synthesized using various methods, as shown in Scheme 1: one-pot (M + M′ + P, Path A), two-step, where a bimetallic amorphous alloy was synthesized first and TOP was added in a second step (M – M′ + P, Path B), and two-step, where a metal phosphide amorphous alloy was synthesized first and the second metal was added in an additional step (M – P + M′, Path C). A 1:1 M/M′ ratio was targeted. In a typical reaction employing Path A, 2–*x* mmol and *x* mmol of the two respective metal precursors involved in the targeted bimetallic phase were added to 2 mL of dried OAm, 3 mL of ODE, and 5 mL of TOP in a 100 mL two-neck Schlenk flask. The reaction sequence for Path A was carried out in a synthesis similar to that of the monometallic species. For reactions carried out using Path B, 2–*x* mmol and *x* mmol of the two respective metal precursors involved in the targeted bimetallic phase were added to 2 mL of dried OAm and 6 mL of ODE in a 100 mL two-neck Schlenk flask. Under constant stirring, the reaction mixture was degassed at 110 °C for 20 min and purged with Ar at 110 °C for 20 min. Once completed, the temperature was increased to 230 °C and allowed to age for 1 h. After 1 h, 5 mL of TOP was injected and the temperature was maintained at 230 °C for an additional 30 min. The temperature was then increased to 300 °C, maintained for 1 h, and cooled naturally to isolate bimetallic phosphide nanoparticles. For reactions carried out using Path C, 2–*x* mmol of one of the metals in the targeted bimetallic phase was added to 2 mL of dried OAm, 3 mL of ODE, and 5 mL of TOP. Under constant stirring, the reaction mixture was degassed at 110 °C for 20 min and purged with Ar at 110 °C for 20 min. The temperature was then increased to 230 °C and aged for 1 h. After 1 h, *x* mmol of the second metal in the targeted phase dissolved in 3 mL of ODE was injected into the reaction mixture. This mixture was aged at 230 °C for 1 h. The temperature was then increased to 300 °C, maintained for 1 h, and cooled naturally to isolate bimetallic phosphide nanoparticles.

### Synthesis of Trimetallic Phosphides (Fe_2–*x*–*y*_Ni_*x*_Co_*y*_P)

Trimetallic phosphide phases were synthesized using similar pathways employed to synthesize bimetallic phosphide phases. In a typical reaction, under the standard conditions in which a 1:1:1 stoichiometry was targeted, the trimetallic phases employed 2–*x*–*y* mmol of Fe precursor, *x* mmol of Ni precursor, and *y* mmol of Co precursor (*x*,*y*,*z* = 0.67). For the compositional variation studies in which the effect of changing the stoichiometry was probed using Scheme 1, Path C, the scale was doubled from 2 to 4 mmol. For example, when a 2:1:1 Fe/Ni/Co ratio was targeted, 2 mmol of Fe(CO)_5_, 1 mmol of Ni(acac)_2_, and 1 mmol of Co(acac)_2_ was employed. Similarly, for the 4:1:1 target ratio, 2.67 mmol of Fe(CO)_5_, 0.67 mmol of Ni(acac)_2_, and 0.67 mmol of Co(acac)_2_ was employed.

### Powder X-ray Diffraction (PXRD)

Powder X-ray diffraction measurements were carried out on a Bruker D2 phaser diffractometer using the Kα line of a Cu anode source. Samples were prepared by placing dried nanoparticles on a zero background SiO_2_ holder, and data were collected in the 2θ = 20–70° range at room temperature. PXRD patterns were compared to the powder diffraction files available in the International Center for Diffraction Data (ICDD) database.

### Transmission Electron Microscopy (TEM) and Energy-Dispersive Spectroscopy (EDS)

Transmission electron micrographs were obtained using a JEOL 2010 electron microscope operating at voltage of 200 kV and beam current of 108 μA. The average dimensions of the nanoparticles and the standard deviation were determined by measuring ∼200 particles using the NIS-Elements D3.10 software and preparing histograms. The electron microscope is equipped with an EDS detector (EDAX Inc.) and was employed to collect compositional data on nanoparticle samples. The compositional data reported in tables throughout the article represent an average of three measurements on three different parts of the grid. The standard deviations are small (typically <5%) and are reported with the average.

### Scanning Transmission Electron Microscopy (STEM)

High-resolution transmission electron micrographs (HRTEM) and EDS maps and line scans were collected using a JEOL-F200 probe-corrected scanning transmission electron microscope operating at 200 kV and equipped with high-angle annular dark-field (HAADF) and EDAX detectors.

### X-ray Photoelectron Spectroscopy (XPS)

X-ray photoelectron spectroscopy measurements were carried out using a NEXSA Thermoscientific X-ray photoelectron spectrometer equipped with a monochromatic Al Kα (1486.7 eV) X-ray source operating at 6 mA and 12 kV. All XPS spectra were fitted using Avantage Software calibrated to the C 1s peak, positioned at 284.8 eV.

## Results and Discussion

To rationalize the syntheses of nanoscale multimetallic phosphides, we first aimed to understand the synthetic pathways enabling formation of monometallic (M_2_P; M = Fe, Co, Ni) and bimetallic (M_2–*x*_M′_*x*_P; M, M′ = Fe, Co, Ni) transition metal phosphide (TMP) systems. Typically, arrested precipitation syntheses of M_2_P (M = Fe, Co, Ni) phases occur via formation of intermediate metal or metal–phosphide amorphous alloy precursor particles at relatively low temperature, followed by crystallization into the desired phosphide phase at higher temperatures.^10,33^ To lay the groundwork in determining how the reactivity of individual precursors affects the formation of bimetallic phosphides, we studied the intermediates and products of single-phase monometallic TMP systems where multivalent metal acetylacetonate (acac) salt or zero-valent metal carbonyl precursors were used in combination with identical amounts of TOP, oleylamine, and octadecene. We chose reaction conditions known to favor formation of M_2_P phases over compositions that are more (e.g., Ni_12_P_5_) or less (e.g., FeP) metal-rich.^8,13,34,35^

### Synthesis of Monometallic Phosphides from Metal Acetylacetonate Precursors

While extremely convenient and less expensive than zero-valent precursors, acac salts need to be chemically reduced to form metal-rich phosphides. An amine (frequently oleylamine) is thus typically employed as a dual-function reagent capable of chemical reduction and surface ligation, although phosphines can themselves act as a reductant, leading in a straightforward manner to acetylacetone and phosphine oxide as byproducts when water is present (see Scheme 3ii) or to acac decomposition products and phosphine oxide in the absence of water.^36^

**Figure 1:**
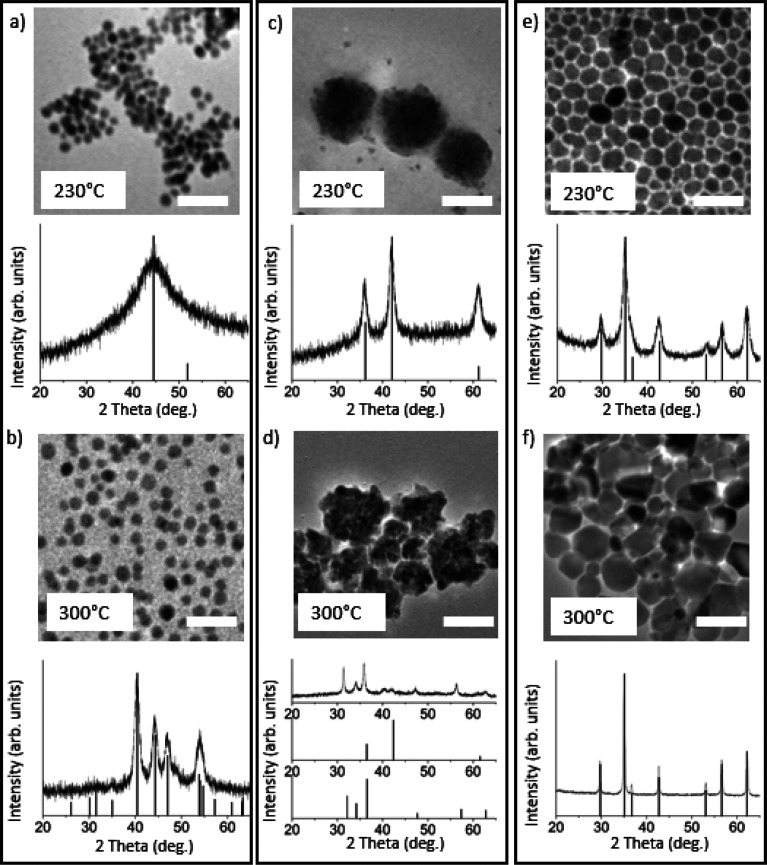
TEM (top) and XRD (bottom) of particles isolated when using (a) Ni(acac)_2_ at 230 °C (fcc-Ni, PDF 000040850), (b) Ni(acac)_2_ at 300 °C (Ni_2_P, PDF 030653544), (c) Co(acac)_2_ at 230 °C (c-CoO, PDF 000481719), (d) Co(acac)_2_ at 300 °C (c-CoO (top), h-CoO (bottom), PDF 010892803), (e) Fe(acac)_3_ at 230 °C, and (f) Fe(acac)_3_ at 300 °C (Fe_3_O_4_, PDF 00190629). Scale bars = 25 nm.

**Scheme 2:**
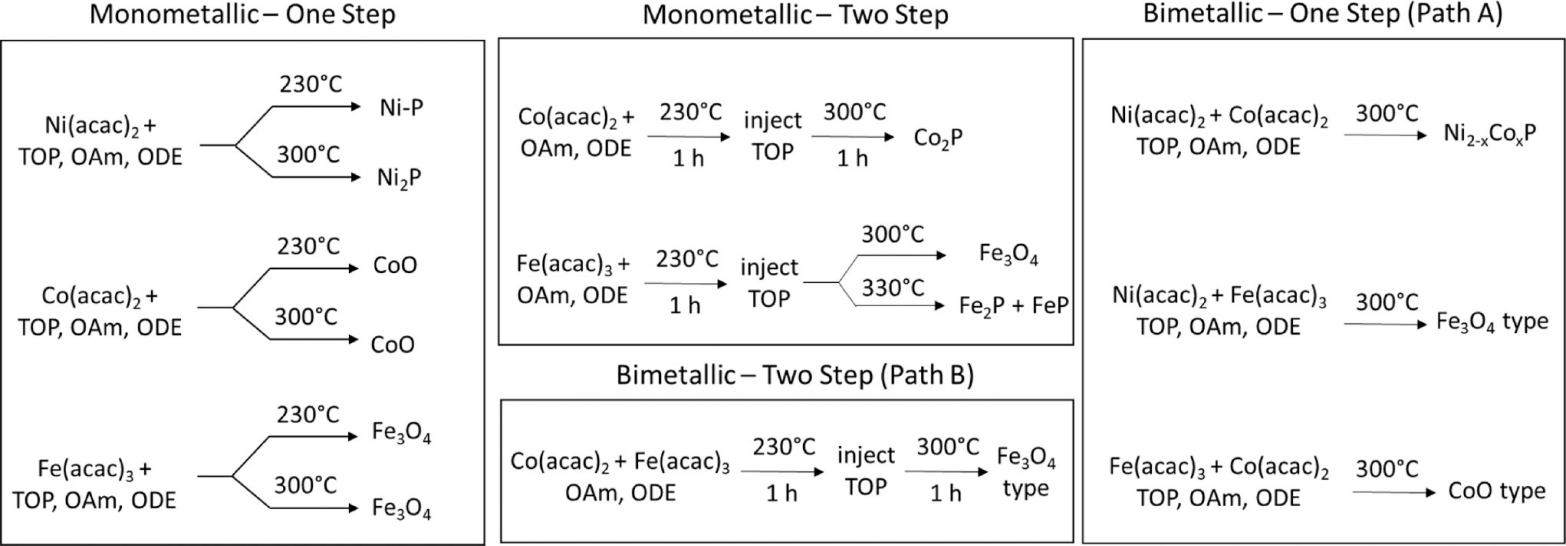
Summary of Reactions with Metal Acetylacetonate Precursors

**Scheme 3:**
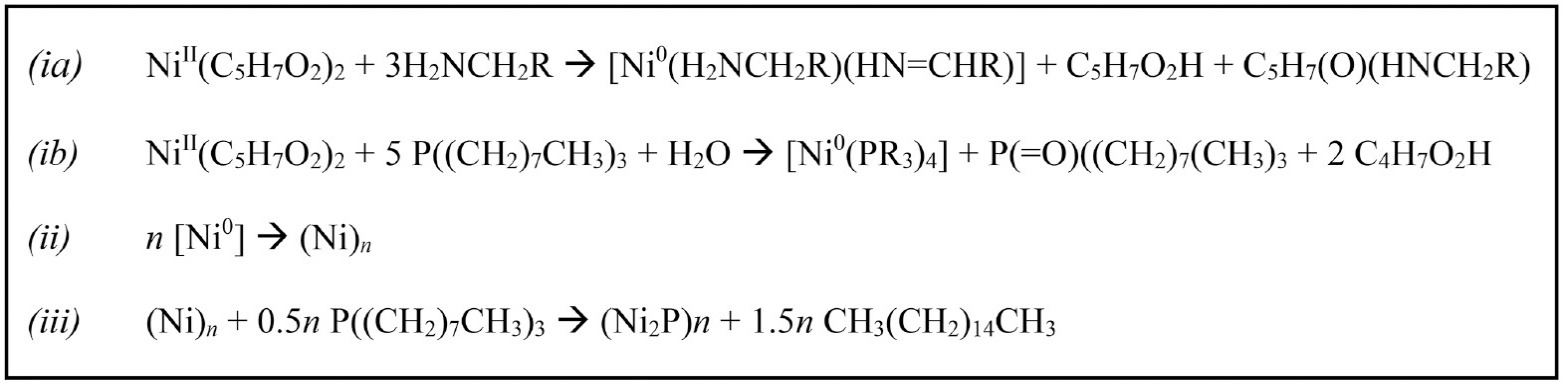
Proposed Series of Reactions for the Synthesis of Ni_2_P Nanoparticles Chemical equations: (i) Reduction of Ni(II) to Ni(0); (ia) via amine coordination to the metal untethering one end of the acac, followed by attack of a second amine equivalent to the (now) pendant carbonyl on the displaced end of the ligand, which subsequently undergoes a condensation reaction to eliminate water and form a bound keto-ene-amine, which is displaced via coordination of a third equivalent of amine that subsequently undergoes β-H elimination to liberate acetylacetone and produce zero-valent Ni coordinated to a bound amine and imine; (ib) via phosphine coordination to form a six-coordinate complex, followed by reaction with water and additional equivalents of phosphine to produce phosphine oxide and acetylacetone as byproducts, and zero-valent Ni coordinated to phosphine ligands; (ii) Ni nanoparticle formation from reaction of zero-valent monomers obtained from Path ia or ib; (iii) phosphidation of Ni nanoparticles, possibly via Ni-catalyzed homolytic bond cleavage and subsequent radical coupling to produce hexadecane.

To evaluate the relative reactivity of acetylacetonate salts of Ni(II), Co(II), and Fe(III) toward phosphidation under reducing conditions, 2 mmol of the relevant salt was independently combined with trioctylphosphine (5 mL, 11.2 mmol), octadecene (3 mL), and oleylamine (2 mL). The mixture was degassed and purged with Ar over 40 min at 110 °C and then heated to either 230 °C (over ∼5 min) or 300 °C (over ∼7 min) for 1 h. The corresponding TEM and PXRD data of the isolated products are shown in Figure 1, and the results are summarized in Scheme 2. Only Ni(acac)_2_ resulted in the formation of the desired M_2_P phase at 300 °C (Figure 1b). The PXRD of the intermediate formed using Ni(acac)_2_ at 230 °C reveals a broad peak stretching over the reference PDF pattern for face-centered cubic (fcc)-Ni, and EDS analysis detected a ∼4.5:1 nickel to phosphorus ratio, suggesting the formation of amorphous Ni–P template particles (Figure 1a and Table 1), as originally reported by Tracy and co-workers.^13,37^ In contrast, when employing Fe(acac)_3_ or Co(acac)_2_, metal oxides (Fe_3_O_4_, CoO) were isolated at both the intermediate and elevated temperature stages (Figure 1c–f), and phosphorus incorporation was minimal (Table 1). These data suggest that Ni(II) is easily reduced by phosphine or oleylamine, whereas Co(II) and Fe(III) are more resistant to reduction. As determined by Carenco and co-workers, the reduction of Ni(acac)_2_ by oleylamine likely proceeds via a ligand-mediated pathway in which reducing equivalents are obtained by conversion of amine to imine (Scheme 3ia). The resultant Ni(0) coordination complexes, [Ni^0^], undergo nucleation and growth to form Ni(0) nanoparticles (Scheme 3ii). Finally, phosphidation of Ni(0) can be rationalized to occur via TOP coordination followed by homolytic P–C bond cleavage and subsequent radical coupling (Scheme 3iii), a mechanism established for gas-phase reactions;^38^ unfortunately, as noted by Carenco et al., mechanistic data on solution-phase reactions involving tertiary alkylphosphines are lacking.^39−42^ Alternatively, as shown in Scheme 3ib, [Ni^0^] can be formed from reducing equivalents resulting from the oxidation of phosphine to phosphine oxide (either from water, as shown in Scheme 3ib, or decomposition of acac).^36^

**Table 1:** EDS (atm %) of Fe, Co, Ni, and P for Products Obtained from the Monometallic Acetyl Acetonate Salt Precursor Reactions Shown in Figure 1

figure	reaction	Fe (atm %)	Ni (atm %)	Co (atm %)	P (atm %)
1a	Ni(acac)_2_ at 230 °C		73.2 ± 0.4		26.8 ± 0.9
1b	Ni(acac)_2_ at 300 °C		63.8 ± 0.7		34.2 ± 0.4
1c	Co(acac)_2_ at 230 °C			99.2 ± 0.3	0.8 ± 0.8
1d	Co(acac)_2_ at 300 °C			97.3 ± 0.7	2.7 ± 1.4
1e	Fe(acac)_3_ at 230 °C	93.1 ± 0.5			6.8 ± 2.7
1f	Fe(acac)_3_ at 300 °C	96.6 ± 0.3			3.4 ± 2.7

Clearly, the solution-phase synthesis of TMP nanoparticles employing metal acetylacetonate precursors is closely associated with their redox reactivity, which is feasible for Ni(acac)_2_ via the proposed sequence of reactions in Scheme 3 but not for Co(acac)_2_ and Fe(acac)_3_ under the chosen conditions of this study. It is challenging to compare the three metals (Ni, Co, Fe) based on redox potentials, as relative redox characteristics depend sensitively on the ligand environment and temperature. This is further complicated by the choice to explore Fe(acac)_3_ when there are other acetylacetonate salt precursors, i.e., Fe(acac)_2_, that may potentially be easier to reduce, as the reduction would be a two-electron process, presumably comparable to Co(II) and Ni(II). In the context of ligand environment, we considered the role of oxophilicity as a proxy for redox potential. That is, a demonstrated preference to bind to oxygen over phosphorus/amine represents a relative oxidation state preference, as metal–phosphorus and metal–amine bonds are more covalent than oxides (and thus functionally have a lower oxidation state). Accordingly, we turned our attention to relative oxophilicities, noting that the three salts all have oxygen donors, and the products formed are either oxides, reduced metals (from reaction with oleylamine or phosphine), or phosphides.^43^ In this context, Ni is unique within the triad in being the least oxophilic (Fe and Co are equally oxophilic), which may explain the relative ease of reduction by amines/phosphines (which are softer than oxides) to form Ni(0) and subsequent phosphidation.

### Targeting Bimetallic Phosphides from Metal Acetylacetonate Precursors Using One-Step Reactions (Scheme 1, Path A)

**Figure 2:**
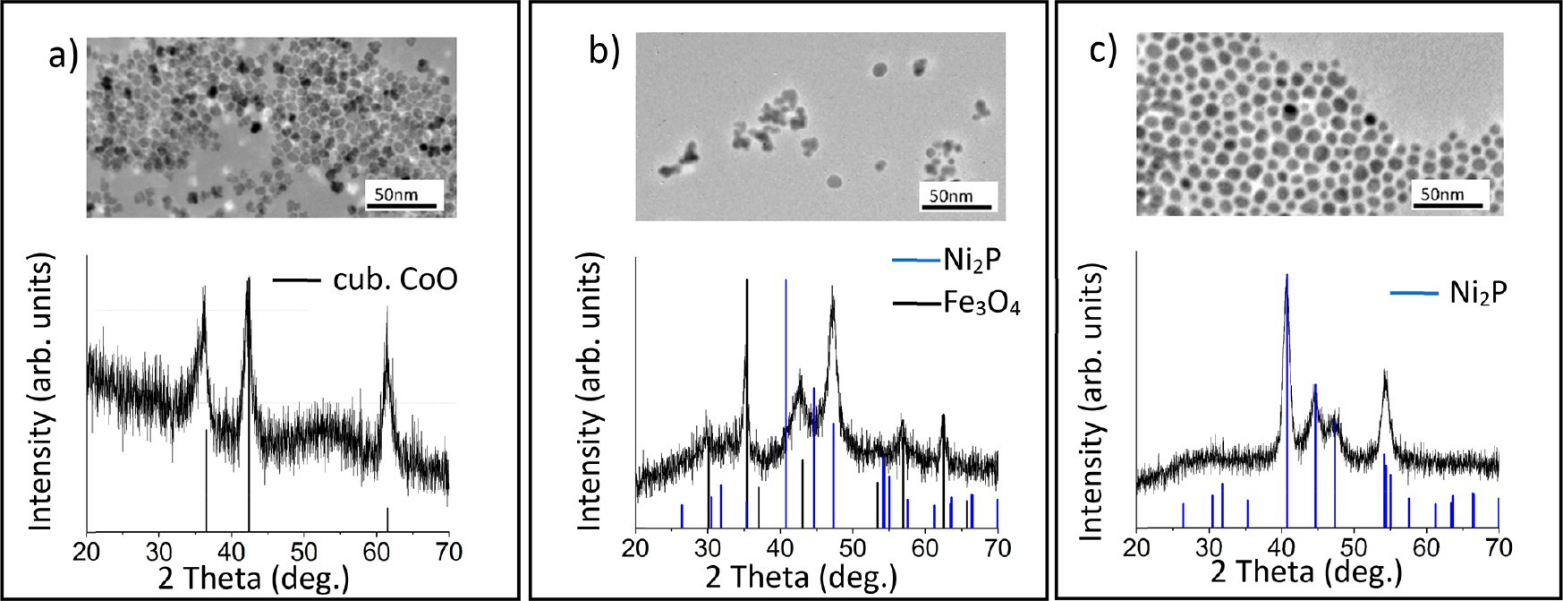
TEM images (top) and PXRD spectra (bottom) of nanoparticles isolated from one step reactions, following Scheme 1, Path A, at 300 °C of (a) Co(acac)_2_ and Fe(acac)_3_, (b) Ni(acac)_2_ and Fe(acac)_3_, and (c) Ni(acac)_2_ and Co(acac)_2_.

Considering the results from the monometallic reactivity studies, when targeting bimetallic phosphides with acac salts, we expect formation of homogeneous bimetallic phases to be difficult with the Fe(acac)_3_ and Co(acac)_2_ precursors if the individual precursors behave independently. However, if Ni can moderate the reduction of Co(II) or Fe(III), bimetallic Ni_2–*x*_M_*x*_P (M = Co, Fe) phases may result. As expected, in one step reactions with the acac precursors the combination of Co(acac)_2_ with Fe(acac)_3_ resulted in formation of oxide nanoparticles that could be indexed to a CoO-type phase (Figure 2a) and very little phosphorus was incorporated (Table 2). Likewise, Ni(acac)_2_ with Fe(acac)_3_ also proved unsuccessful, forming a combination of an Fe_3_O_4_-type oxide with an M_2_P phase, likely Ni_2_P (Figure 2b and Table 2). However, a one-step reaction employing Ni(acac)_2_ and Co(acac)_2_ resulted in solid solution formation of the bimetallic Ni_2–*x*_Co_*x*_P phase (Figure 2c). The isolated nanoparticles were spherical in morphology (diameter = 8.3 ± 1.1 nm) with an EDS atomic Ni/Co ratio of ∼1:1 and a 60/40 ratio of metal to phosphorus (Table 2). We hypothesize that the success of this reaction is due to reduction of Co(acac)_2_ mediated by reduced nickel (Ni(0) or Ni–P), enabling the formation of Ni_2–*x*_Co_*x*_P, as OAm or TOP does not reduce Co(acac)_2_ under these conditions.

**Table 2:** EDS (atm %) of Fe, Co, Ni, and P for the Products of the Bimetallic Acetylacetonate Salt Precursor Reactions Shown in Figure 2

figure	reaction	Fe (atm %)	Ni (atm %)	Co (atm %)	P (atm %)
2a	Path A: Co(acac)_2_ + Fe(acac)_3_	45.9 ± 0.8		52.3 ± 0.8	1.8 ± 1.5
2b	Path A: Ni(acac)_2_ + Fe(acac)_3_	26.4 ± 0.4	46.6 ± 0.3		27.0 ± 0.4
2c	Path A: Ni(acac)_2_ + Co(acac)_2_		30.6 ± 0.8	28.9 ± 0.7	40.5 ± 0.6

### Targeting Bimetallic Phosphides from Metal Acetylacetonate Precursors Using Two-Step Reactions (Scheme 1, Path B)

To better understand the reduction process for acac salts, we sought to decouple reduction by oleylamine from phosphidation by adding trioctylphosphine in a second step, as shown in Scheme 2 (Monometallic – Two Step) and described in the Supporting Information S1. We found that under suitably high temperatures, Co(acac)_2_ (300 °C) and Fe(acac)_3_ (330 °C) can lead to the corresponding phosphides (Figures S1 and S2), albeit via an oxide intermediate. In this case, reduction via phosphine likely occurs by oxygen atom abstraction from the metal, and phosphidation occurs with further equivalents of phosphine. In this context, it is not clear why CoO reduction and phosphidation to Co_2_P is observed in the two-step reaction, but not in the one-step. However, surface area may play a role in facilitating the two-step reaction, since the majority of particles formed in the two-step reaction are small and disperse (∼8 nm, Figure S1), whereas ∼25 nm supraparticles are formed in the one-step reaction (Figure 1d). Despite success with monometallic phosphide formation, attempts to synthesize bimetallic phosphides by a two-step process in which reduction is attempted first, followed by phosphidation (Scheme 2, Bimetallic – Two Step), proved unsuccessful for Fe–Co and Fe–Ni (Supporting Information S2, Figure S3 and Table S1), producing oxide phases in all cases. These studies suggest that, in general, Ni(acac)_2_ is a suitable precursor toward the formation of monometallic and bimetallic metal phosphides, Co(acac)_2_ can be useful but challenging, and Fe(acac)_3_ is a poor choice. Thus, other metal precursors for Co and Fe need to be considered.

### Targeting Monometallic Phosphides from Metal Carbonyl Precursors: Co, Fe

**Scheme 4:**
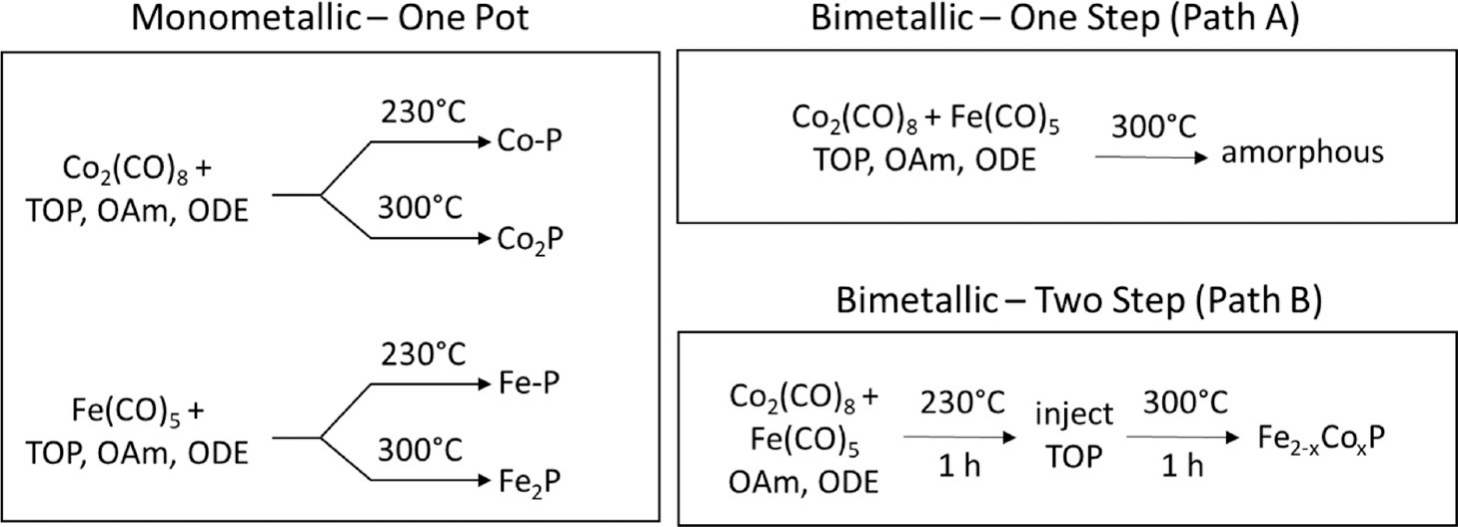
Summary of Reactions with Metal Carbonyl Precursors

**Figure 3:**
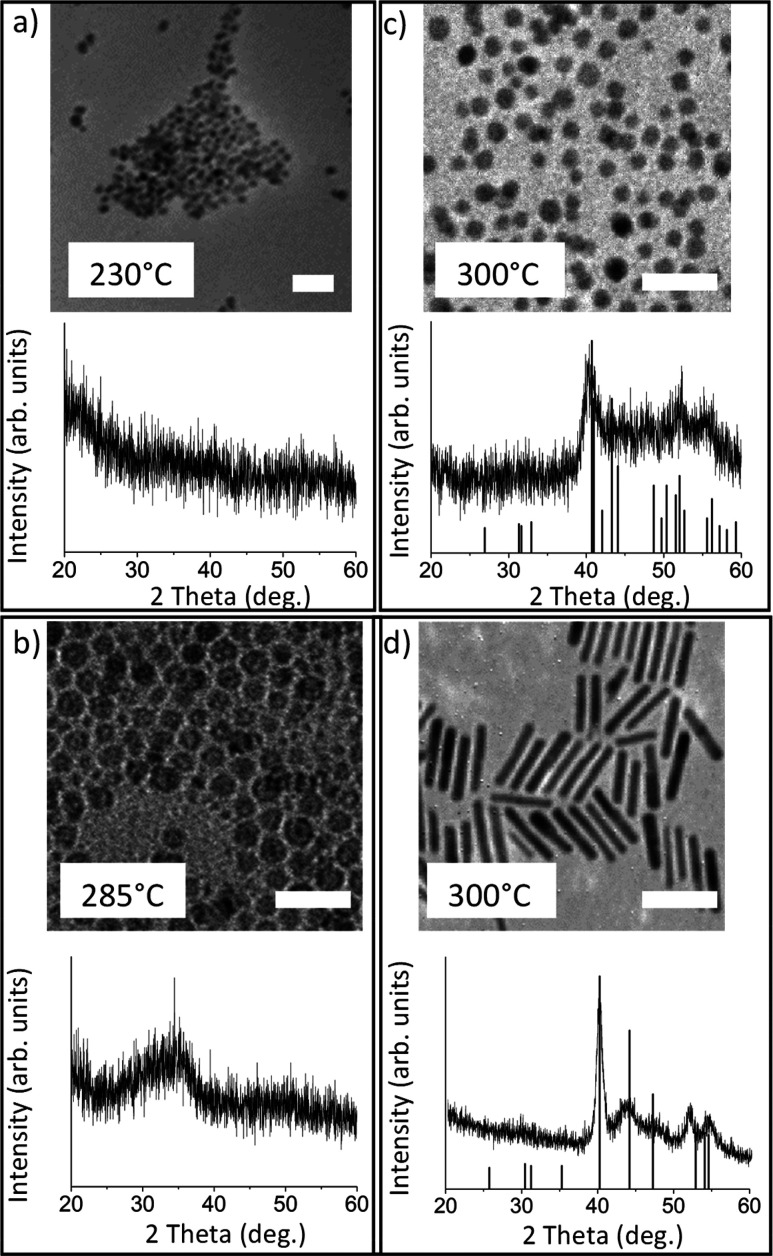
TEM images (top) and PXRD (bottom) of nanoparticles isolated when using (a) Co_2_(CO)_8_ at 230 °C, (b) Fe(CO)_5_ at 285 °C, (c) Co_2_(CO)_8_ at 300 °C (Co_2_P PDF), and (d) Fe(CO)_5_ at 300 °C (Fe_2_P PDF). Scale bars = 25 nm.

As a means to address the challenges faced by reducing Co and Fe acac precursors in the monometallic reactions, we turned our attention to zero-valent metal carbonyls as a source for these metals, using the same reaction ratios, solvents, and temperature parameters. The reaction parameters and data are summarized in Scheme 4, and the TEM and PXRD data are shown in Figure 3. When Co_2_(CO)_8_ was employed, small spherical particles, ∼3–5 nm, were isolated at the intermediate temperature, 230 °C (Figure 3a). Although the PXRD is featureless, EDS analysis reveals a Co/P atomic ratio of ∼59:41, suggesting amorphous Co–P formation (Table 3). In contrast, Fe(CO)_5_ was unreactive at 230 °C; the solution remained bright yellow, indicating no nucleation events have taken place. A color change to black was noted approaching 285 °C, and thus we isolated the “intermediate phase” after heating at 285 °C for 1 h. As shown in Figure 3b, the particles are spherical and polydisperse, ranging in size from ∼7.5 to 9.6 nm. The PXRD revealed an amorphous diffraction pattern, and EDS analysis detected ∼17% incorporation of P, suggesting the formation of an Fe–P amorphous alloy intermediate (Table 3). When heated to 300 °C, Co_2_P (Figure 3c) and Fe_2_P (Figure 3d) phases were isolated in each case, according to PXRD data, the former as spherical particles of size 5.6 ± 0.9 nm and the latter as nanorods of dimension 30.7 ± 5.2 nm × 7.0 ± 1.2 nm. EDS data (Table 3) are consistent with excess P in each sample, reducing the expected 2:1 ratio of M/P to 1.9:1 for Co/P and 1.7:1 for Fe/P. This may be attributed to a minor impurity phase not evident from PXRD (e.g., CoP, FeP), or residual TOP on the particle surface. Overall, these data suggest a pathway whereby amorphous Co–P is formed initially upon temperature ramping and undergoes complete conversion and crystallization to the desired M_2_P phase at higher temperatures, whereas Fe–P particles are formed en route to the formation of the desired Fe_2_P phase, but higher temperatures than 230 °C are necessary to thermally decompose Fe(CO)_5_. Determining how the individual metal acetylacetonate salt and metal carbonyl precursors behave under our chosen reaction parameters and solvent system allows us to establish the necessary conditions to pursue the trimetallic Fe_2–*x*–*y*_Ni_*x*_Co_*y*_P phase discussed in the latter half of this study.

**Table 3:** EDS (atm %) of Fe, Co, and P for the Monometallic Metal Carbonyl Precursor Reactions Shown in Figure 3

figure	reaction	Fe (atm %)	Co (atm %)	P (atm %)
3a	Co_2_(CO)_8_ at 230 °C		59.0 ± 0.8	41.0 ± 1.1
3b	Fe(CO)_5_ at 285 °C	82.5 ± 0.7		17.5 ± 1.8
3c	Co_2_(CO)_8_ at 300 °C		65.3 ± 1.2	34.7 ± 2.1
3d	Fe(CO)_5_ at 300 °C	63.2 ± 2.0		36.8 ± 2.1

### Kinetics of Phosphidation of Fe(CO)_5_, Co_2_(CO)_8_, and Ni(acac)_2_

**Scheme 5:**
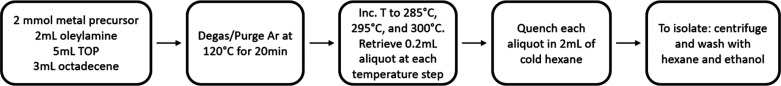
Reaction Scheme Describing How Aliquot Studies Were Carried Out

**Figure 4:**
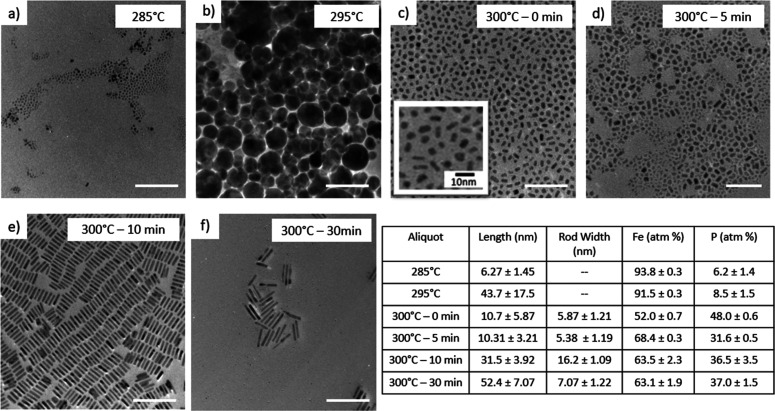
TEM of aliquots isolated when using Fe(CO)_5_ in one step reaction at (a) 285 °C, (b) 295 °C, (c) 300 °C – 0 min, (d) 300 °C – 5 min, (e) 300 °C – 10 min, and (f) 300 °C – 30 min. Scale bars = 100 nm. The inset table shows EDS ratios (Fe/P) and size distribution data corresponding to panels a–f.

The relative kinetics of phosphidation and crystallization is important to determine the order of addition when considering the synthesis of multimetallic phases, that is, whether to pursue Path A, B, or C, according to Scheme 1. To probe the relative reactivity of the metal precursor with the reaction components for the precursors that successfully formed the desired monometallic phase, aliquot studies were performed with respect to temperature and time using Fe(CO)_5_, Co_2_(CO)_8_, and Ni(acac)_2_, as shown in Scheme 5. For the Fe(CO)_5_ study, TEM and EDS data are shown in Figure 4 (the amounts isolated in each aliquot were insufficient for PXRD analysis). In the case of Fe(CO)_5_, the aliquot retrieved at 285 and 295 °C is mostly composed of iron with little phosphorus incorporation, forming very small particles initially (several nanometers at 285 °C), that grow rapidly to several tens of nanometers at 295 °C (Figure 4a,b). At 300 °C, 0 min, there is the abrupt uptake of phosphorus, with the Fe/P ratio leveling out at nearly 1:1 (Figure 4 inset table, Figure 4c). The nanoparticles retrieved at this temperature and time consist of a mixture of oblong spheres and short rods. After 5 min at 300 °C, the composition begins to reflect the desired binary metal phosphide phase with the Fe/P ratio of 2:1 (Figure 4d). Formation of the characteristic nanorod morphology for Fe_2_P phases occurs at 300 °C, 10 min, and the nanorods grow in length (up to 52.36 ± 7.07 nm) as the reaction time progresses (Figure 4e).

**Figure 5:**
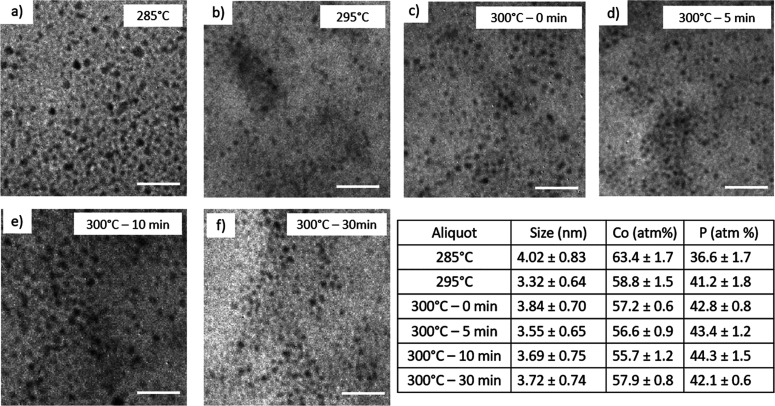
TEM of aliquots isolated when using Co_2_(CO)_8_ in one-step reaction at (a) 285 °C, (b) 295 °C, (c) 300 °C – 0 min, (d) 300 °C – 5 min, (e) 300 °C – 10 min, and (f) 300 °C – 30 min. Scale bars = 50 nm. The inset table shows EDS ratios (Co/P) and size distribution data corresponding to panels a–f.

In contrast to Fe(CO)_5_ the uptake of phosphorus is much faster in the case of Co_2_(CO)_8_, with the first aliquot retrieved at 285 °C having over 36% phosphorus incorporation (Figure 5a). As temperature and time were increased, the Co/P ratio leveled out to ∼1.3:1 and particles remained spherical in shape with average diameters between 3.32 and 3.72 nm (Figure 5b–f). Relative to Fe(CO)_5_, the aliquot studies with Co_2_(CO)_8_ reveal that significant phosphidation occurs at much lower temperature and time stages (Figure 5, inset table). The excess phosphorus detected suggests CoP is a competing byproduct here, which is also a reflection of the ease of phosphidation.^10^

**Figure 6:**
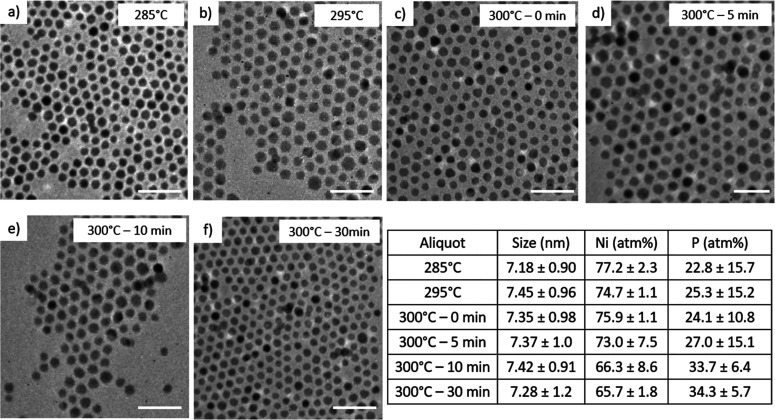
TEM of aliquots isolated when using Ni(acac)_2_ in one-step reaction at (a) 285 °C, (b) 295 °C, (c) 300 °C – 0 min, (d) 300 °C – 5 min, (e) 300 °C – 10 min, and (f) 300 °C – 30 min. Scale bars = 25 nm. The inset table shows EDS ratios (Ni/P) and size distribution data corresponding to panels a–f.

Similar to Co_2_(CO)_8_, the rate of phosphidation when employing Ni(acac)_2_ is much faster than with Fe(CO)_5_ (Figure 6). Due to the facile reduction of Ni(II) and subsequently increased nucleation, we were also able to isolate enough samples from the Ni(acac)_2_ reactivity series aliquots to run PXRD measurements (Figure S4). The first aliquot retrieved at 285 °C had a Ni/P ratio of ∼3.4:1, and the particles are spherical in shape with a diameter of 7.18 ± 0.90 nm (Figure 6a). Looking at the PXRD pattern of the particles isolated at 285 °C, it is apparent that Ni_2_P formation had already started with distinct reflections that can be identified as the (111), (201), and (210) Ni_2_P planes (Figure S4). The excess Ni detected from the first aliquot can be attributed to the metal rich Ni_7_P_3_ species also present as observed by PXRD (Figure S4). The reflections belonging to the detected Ni_7_P_3_ species subside as temperature and time increases, suggesting the transformation from a more metal rich species to the desired M_2_P phase (EDS ratios also support this observation, Figure 6). The aliquot at 300 °C, 10 min, matches well with the PXRD pattern for phase-pure Ni_2_P, and the particles are nearly monodisperse with spherical morphology (Figure 6e). In the aliquot retrieved at 300 °C, 10 min, we begin to see the Ni/P ratio level out to 2:1, reflecting the crystallization of the desired Ni_2_P phase (Figure 6f). From the monometallic rate of phosphidation studies, it was observed that the formation of crystalline Ni_2_P particles using Ni(acac)_2_ began as early as 285 °C (Figure S4) and the uptake of P using Co_2_(CO)_8_ is comparable to this system, whereas with Fe(CO)_5_ phosphidation is minimal below the crystallization temperature of 300 °C (see table insets, Figures 4−6). We note that the apparent sharp uptake of P noted at 300 °C (0 min) is unlikely to be instantaneous and likely reflects a combination of additional time (10 min) and gradually increasing temperature from the prior set point (295 °C). These aliquot studies distinguish the relative rate of phosphidation between precursors, which becomes an important consideration in accessing the trimetallic Fe_2–*x*–*y*_Ni_*x*_Co_*y*_P phase later in this study.

### Synthesis of Bimetallic Phosphides: Ni_2–*x*_Co_*x*_P

**Figure 7:**
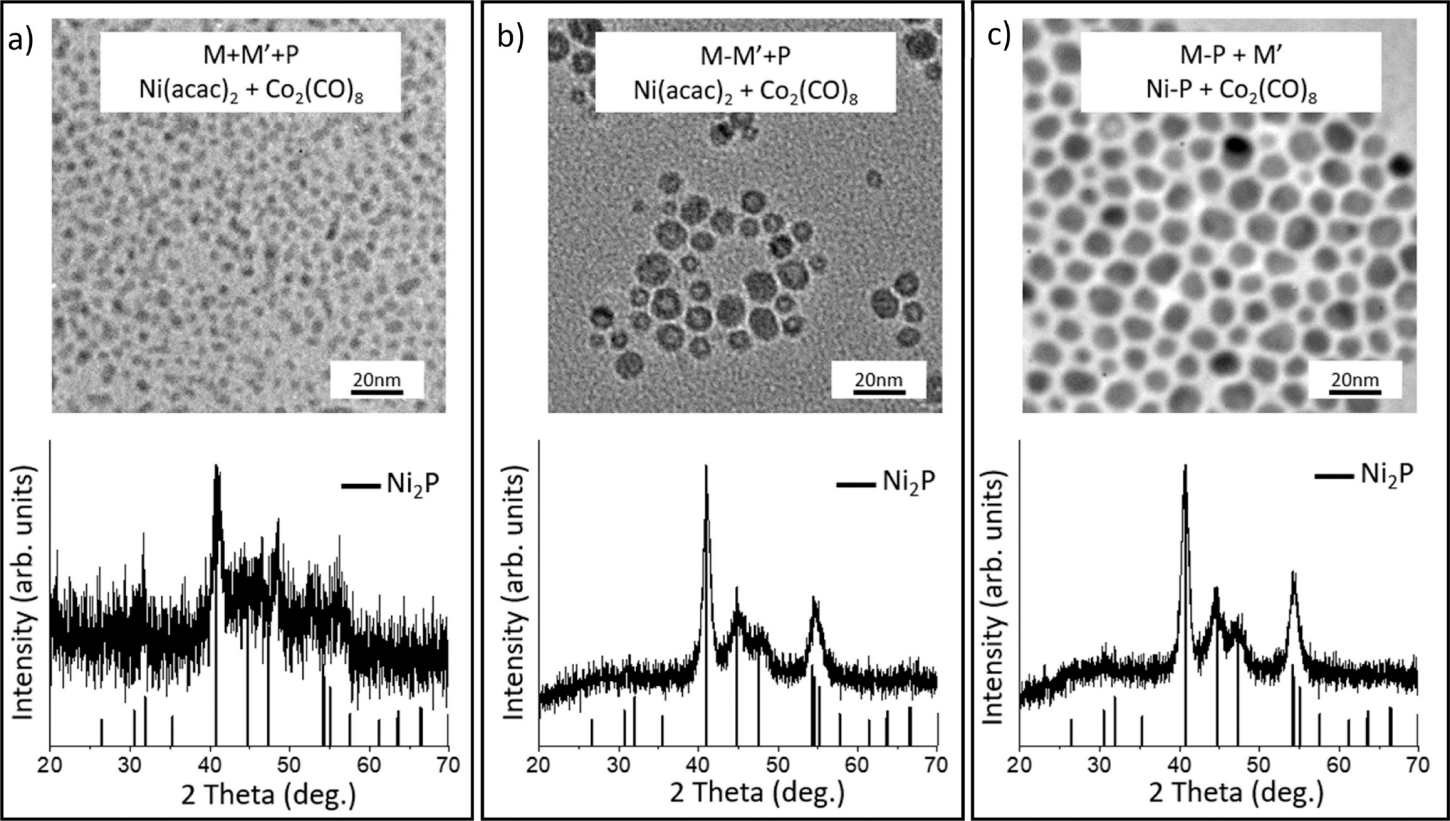
PXRD and TEM of (a) Path A: one-step reaction with Ni(acac)_2_ and Co_2_(CO)_8_ at 300 °C; (b) Path B: a two-step reaction where a Ni–Co amorphous alloy was synthesized first at 230 °C and TOP is added in second step, crystallization *T* = 300 °C; (c) Path C: a two-step reaction where Ni–P amorphous alloy was synthesized in the first step at 230 °C and Co_2_(CO)_8_ is added in a subsequent step, crystallization *T* = 300 °C. The diffraction pattern of Ni_2_P (PDF 030653544) is shown for comparison.

Based on the relative rate of phosphidation studies, it can be predicted that Ni_2–*x*_Co_*x*_P nanoparticles can be isolated through a simple combination of Ni(acac)_2_ and Co_2_(CO)_8_ in a one-step reaction with ODE, OAm, and TOP (Scheme 1, Path A). When this reaction is carried out, it is observed that the collected PXRD pattern of the isolated nanoparticles matches well with the PDF pattern for Ni_2_P (Figure 7a). The TEM image reveals nanoparticles that are spherical in morphology with sizes of ∼4 nm and EDS Ni/Co ratios of 1:1 (Table 4). When Scheme 1, Path B, is employed in which a bimetallic amorphous alloy is synthesized initially at 230 °C and TOP is injected in an additional step, the collected PXRD spectra matched well with Ni_2_P phase formation and the metal ratios again have the target 1:1 Ni/Co stoichiometry (Figure 7b and Table 4). Particles synthesized via this method have hollow morphology with diameters of approximately 8.6 ± 2.1 nm. We presume this is a reflection of the Kirkendall effect (faster diffusion of Ni and Co outward, than P inward), but the core–shell morphology associated with NiCo bimetallic alloys may also be a contributing factor.^44−46^ Utilizing Scheme 1, Path C, with these precursors, a Ni_2–*x*_Co_*x*_P solid solution was isolated with the PXRD patterns matching with the reference pattern for Ni_2_P (Figure 7c). TEM and EDS revealed particles with spherical morphology (diameter ∼8.8 ± 1.3 nm) and chemical composition of Ni_1.1_Co_0.9_P_1.1_, respectively. The excess Ni is likely a consequence of the prealloying step in which Ni–P is formed. Longer overall reaction times may be needed to achieve comparable incorporation of Co. Clearly, the Ni–Co–P system is relatively well behaved, with each of the pathways tested being viable protocols to access solid solutions of the bimetallic phase, albeit with significant differences in size and polydispersity. Furthermore, these findings suggest that when attempting to synthesize homogeneous trimetallic nanoparticles, routes that go through a Ni–Co–P intermediate may be a viable pathway.

**Table 4:** EDS (atm %) of Ni, Co, and P and Average Particle Size from TEM Analysis for the Bimetallic Ni_2–*x*_Co_*x*_P Reactions Employing Ni(acac)_2_ and Co_2_(CO)_8_

figure	reaction	Ni (atm %)	Co (atm %)	P (atm %)	size (nm)
7a	Path A: Ni + Co + P	29.9 ± 0.9	28.9 ± 0.9	41.2 ± 0.7	2.9 ± 0.5
7b	Path B: Ni–Co + P	29.5 ± 1.2	29.6 ± 1.2	40.9 ± 1.1	8.6 ± 2.1
7c	Path C: Ni–P + Co	36.6 ± 0.3	28.7 ± 0.3	34.7 ± 0.3	8.8 ± 1.3

### Synthesis of Bimetallic Phosphides: Fe_2–*x*_Ni_*x*_P

**Figure 8:**
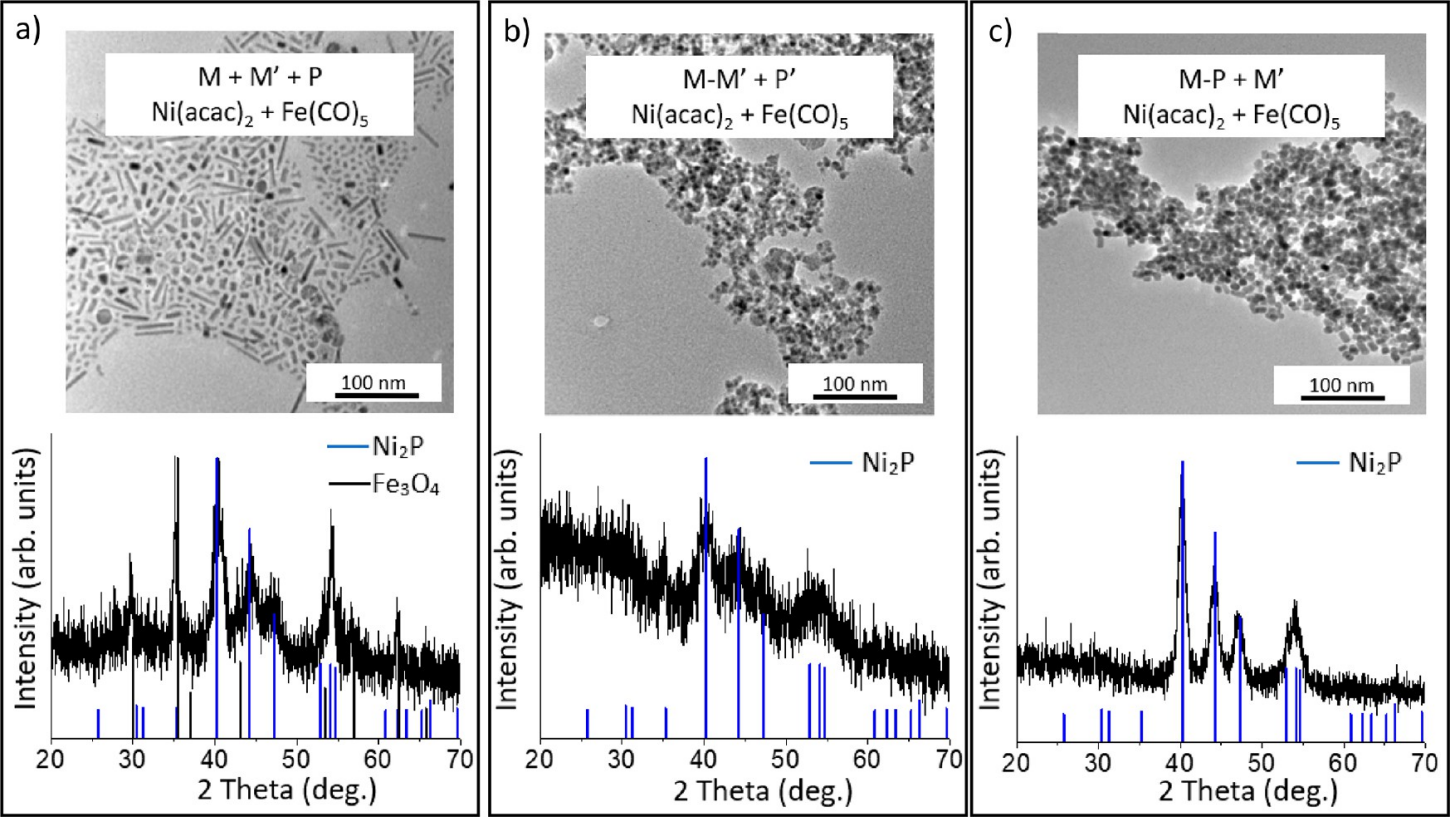
PXRD and TEM of (a) Path A: one-step reaction with Fe(CO)_5_ and Ni(acac)_2_ at 300 °C; (b) Path B: a two-step reaction where Fe–Ni amorphous alloy was synthesized in the first step at 230 °C and TOP is added in a subsequent step, crystallization *T* = 300 °C; (c) Path C: a two-step reaction where Ni–P amorphous alloy was synthesized in the first step at 230 °C and Fe(CO)_5_ is added in a subsequent step, crystallization *T* = 300 °C. Reference patterns for Fe_3_O_4_ and are provided for comparison.

Using what we learned from the relative reactivity studies, we predict that when making Fe_2–*x*_Ni_*x*_P with Fe(CO)_5_ and Ni(acac)_2_, due to the large difference in the relative phosphidation rates of these two metal precursors, a one-step slow-heating reaction (Scheme 1, Path A) should result in phase segregation and insufficient conversion to the desired Fe_2–*x*_Ni_*x*_P phase. Indeed, the PXRD data from one-step reactions with Fe(CO)_5_, Ni(acac)_2_, and TOP indicate a phase-segregated product matching the reference patterns of Fe_3_O_4_ and Ni_2_P (Figure 8a). The TEM images revealed two types of morphologies: irregular spheres (diameter ∼9.8 ± 3.6 nm) and polydisperse rods (*L* = 40.4 ± 10.8 nm, *W* = 4.9 ± 1.2 nm), which further supports the presence of phase segregation. In the bimetallic phase, the differences in phosphidation rates combined with the quick decomposition of the metal acac precursor potentially serves as an adventitious source of oxygen, encouraging the formation of iron oxide byproducts and phase-segregated materials. These data suggest that metal chloride precursors, often employed in transition metal chalcogenide synthesis, may be better suited to synthesize TMP nanomaterials without oxide formation.^47,48^

To mediate the differences in reactivity between the metal precursors and individual reaction components we employed a prealloying step, according to Scheme 1, Path B. An Fe–Ni amorphous alloy is synthesized first and then TOP is added in an additional step, yielding a M_2_P product with an XRD pattern matching with the reference pattern for Ni_2_P (Figure 8b). The Fe_2–*x*_Ni_*x*_P particles isolated using this pathway have irregular spherical morphology (diameter 4.6 ± 1.0 nm) and the composition slightly varies from the target 1:1 ratio (Table 5). In contrast, when employing Scheme 1, Path C, we find that making a Ni–P amorphous alloy first and introducing Fe(CO)_5_ in a second step results in crystalline Fe_2–*x*_Ni_*x*_P nanoparticles, with characteristic short-rod morphology. Moreover, the composition more closely reflects the 1:1:1 Fe/Ni/P target ratio when this pathway is employed (Figure 8c and Table 5).

**Table 5:** EDS (atm %) of Fe, Ni, and P and Average Particle Size from TEM Analysis for the Bimetallic Fe_2–*x*_Ni_*x*_P Reactions Employing Fe(CO)_5_ and Ni(acac)_2_

figure	reaction	Fe (atm %)	Ni (atm %)	P (atm %)	length (nm)	width (nm)
8a	Path A: Ni + Fe + P	18.7 ± 1.7	40.1 ± 1.0	41.3 ± 1.0	40.4 ± 10.8	4.9 ± 1.2
8b	Path B: Ni–Fe + P	39.6 ± 0.5	20.5 ± 0.8	39.9 ± 0.5	4.6 ± 1.0	
8c	Path C: Ni–P + Fe	35.4 ± 0.5	30.7 ± 0.6	33.9 ± 0.6	12.6 ± 1.9	8.5 ± 1.3

### Synthesis of Bimetallic Phosphides: Fe_2–*x*_Co_*x*_P

**Figure 9:**
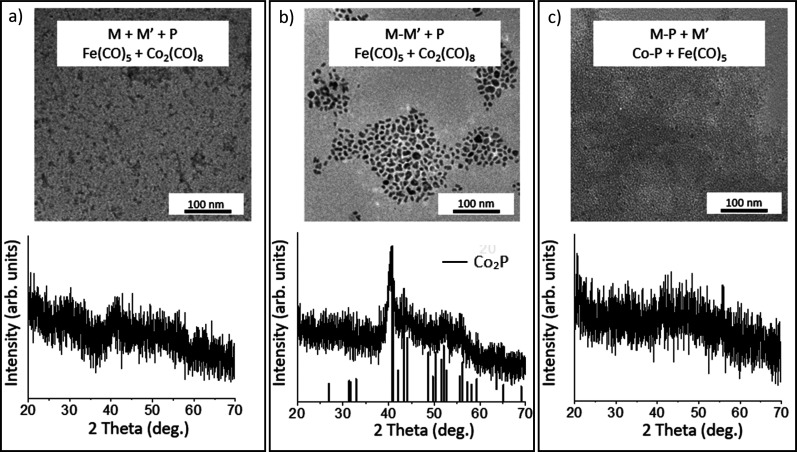
PXRD and TEM of (a) Path A: one-step reaction with Fe(CO)_5_ and Co_2_(CO)_8_ at 300 °C; (b) Path B: a two-step reaction where Fe–Co amorphous alloy was synthesized in the first step at 230 °C and TOP is added in a subsequent step, crystallization *T* = 300 °C; (c) Path C: a two-step reaction where Co–P amorphous alloy was synthesized in the first step at 230 °C and Co_2_(CO)_8_ is added in a subsequent step, crystallization *T* = 300 °C. The reference pattern for Co_2_P is provided for comparison.

The clear differences in phosphidation between Fe(CO)_5_ and Co_2_(CO)_8_ determined by the relative kinetics of phosphidation studies suggest that a two-step protocol would be necessary to form the bimetallic phosphide phase, similar to Fe_2–*x*_Ni_*x*_P. A one step reaction with Fe(CO)_5_ and Co_2_(CO)_8_ resulted in tiny nanoparticles (diameter 4.7 ± 0.9 nm) with a corresponding featureless PXRD patterns (Figure 9a). In contrast, Scheme 1, Path B, shows that a bimetallic Fe–Co amorphous alloy is synthesized initially and TOP is added in a second step, providing a direct pathway for synthesizing Fe_2–*x*_Co_*x*_P (Figure 9b). The isolated nanoparticles have a hollow sphere morphology with an average diameter of 9.6 ± 2.5 nm, as a result of the Kirkendall effect, consistent with literature reports of this phase.^45,49^ Intriguingly, this reaction also leads to a crystalline M_2_P phase when the Co(acac)_2_ precursor is combined with Fe(CO)_5_ (Figure S3c), although the samples are quite polydisperse and cobalt-rich (Table S1). We also tried this reaction according to Scheme 1, Path C, where a Co–P amorphous alloy is synthesized initially and Fe(CO)_5_ is added in a subsequent step. Small 2.6 ± 0.6 nm nanospheres with a relatively featureless PXRD pattern (Figure 9c) result. Notably, the P atm % is superstoichiometric in all cases, with M/P values of nearly 1:1 (Table 6). Based on previous studies, we expect that the M/P ratio can be increased by increasing the oleylamine to TOP ratio in the synthesis to favor metal incorporation.^13,50^

**Table 6:** EDS (atm %) of Fe, Co, and P and Average Particle Sizes from TEM for the Bimetallic Fe_2–*x*_Co_*x*_P Reactions Employing Fe(CO)_5_ and Co_2_(CO)_8_

figure	reaction	Fe (atm %)	Co (atm %)	P (atm %)	size (nm)
9a	Path A: Fe + Co + P	31.1 ± 2.7	22.6 ± 3.0	46.3 ± 1.8	4.7 ± 0.9
9b	Path B: Fe–Co + P	22.6 ± 0.5	26.7 ± 0.5	50.7 ± 0.3	9.6 ± 2.5
9c	Path C: Co–P + Fe	19.2 ± 1.6	28.8 ± 1.0	52.0 ± 0.8	2.6 ± 0.6

### Synthesis of Trimetallic Fe_2–*x*–*y*_Ni_*x*_Co_*y*_P Nanoparticles

A summary of the results for synthesis of bimetallic phosphides according to the three different pathways, and as a function of precursor, is shown in Table 7. Based on our study of the bimetallic systems, we can conclude that the Ni–Co system produces crystalline bimetallic phosphides under all the conditions we tested (and independent of Co precursor selection, Co_2_(CO)_8_ or Co(acac)_2_), while the Fe–Ni system prefers Path B (Fe–Ni + P) or Path C (Ni–P + Fe) using Ni(acac)_2_ and Fe(CO)_5_. The Fe–Co system is successful when a bimetallic amorphous alloy is formed in step 1 (Path B) from Fe(CO)_5_ and Co_2_(CO)_8_ (or Co(acac)_2_), or a cobalt–phosphorus amorphous alloy is formed in step 1 from Co_2_(CO)_8_ (Path C), and Fe(CO)_5_ is added in a subsequent step (note that we did not explicitly test mixtures of Co(acac)_2_ and Fe(CO)_5_ precursors by Path C, as phosphidation of Co(acac)_2_ under our reaction conditions leads to oxide in the absence of Ni. From these studies, it is evident that a prealloying step will be critical to accessing new trimetallic Fe_2–*x*–*y*_Ni_*x*_Co_*y*_P TMP phases while employing these precursors.

**Table 7:**
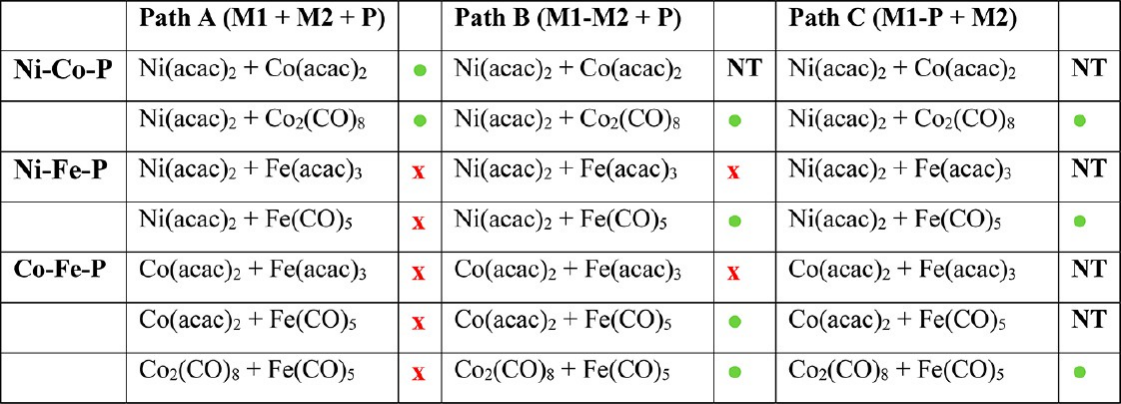
Summary of Results for Syntheses of Bimetallic Phosphides of Ni, Co, and Fe as a Function of Precursor and Synthetic Pathway^a^

a NT = reaction not tried; green circle = reaction produced crystalline bimetallic phosphide; red x = reaction produced oxide or was amorphous.

Based on our analysis, the most reasonable approach to make the ternary phase under these reaction conditions and solvent system would be to either make the Ni–Co–Fe amorphous alloy first, and then phosphide (Scheme 1, Path B) or make the Ni–Co–P amorphous alloy first and add Fe in a subsequent step, as a modification of Scheme 1, Path C. Moreover, because Co(acac)_2_ is active when Ni(acac)_2_ is present, we hypothesized that we would be able to prepare the trimetallic phases without having to resort to the Co_2_(CO)_8_ precursor.

#### Targeting Fe_0.67_Ni_0.67_Co_0.67_P

We first tested our presumption that a single-step reaction via Scheme 1, Path A, would not be successful. As shown in Figure S5a, combining Ni(acac)_2_, Co(acac)_2_, and Fe(CO)_5_ in a 1:1:1 ratio with TOP at the outset resulted in a combination of oxide and phosphide phases based on PXRD. In the TEM images, both nanorods and irregular spheres are present, further indicating formation of inhomogeneous phases (Figure S5b,c). EDS mapping reveals phase segregation with Fe, Ni, Co, and P being unevenly distributed throughout the morphological features present (Figure S5d–g). We also tried Scheme 1 Path A, with Co_2_(CO)_8_ in lieu of Co(acac)_2_ to see if the Co source would make a difference (Figure S6a). In this case, very large aggregates were obtained, but the product was amorphous and compositionally iron rich (Fe/Ni/Co = 2.2:1.0:1.2), Table S2.

We next considered a two-step procedure, Scheme 1, Path B, in which the three metal precursors (Ni(acac)_2_, Co(acac)_2_, and Fe(CO)_5_) are alloyed together, followed by phosphidation. As shown in Figure S6c, the particles formed are small and spherical in shape, 10.2 ± 1.2 nm, and the PXRD pattern is consistent with M_2_P phase formation, an encouraging sign. However, the sample is quite iron-rich, with a Fe/Ni/Co ratio of 2.1:1.0:1.1 (Table S2). Substitution of Co(acac)_2_ with Co_2_(CO)_8_ results in polydisperse samples with larger, hollow particles (Figure S6b), but the stoichiometry is, once again, iron-rich (Fe/Ni/Co ratio = 2.3:1.0:1.6; Table S2).

**Figure 10:**
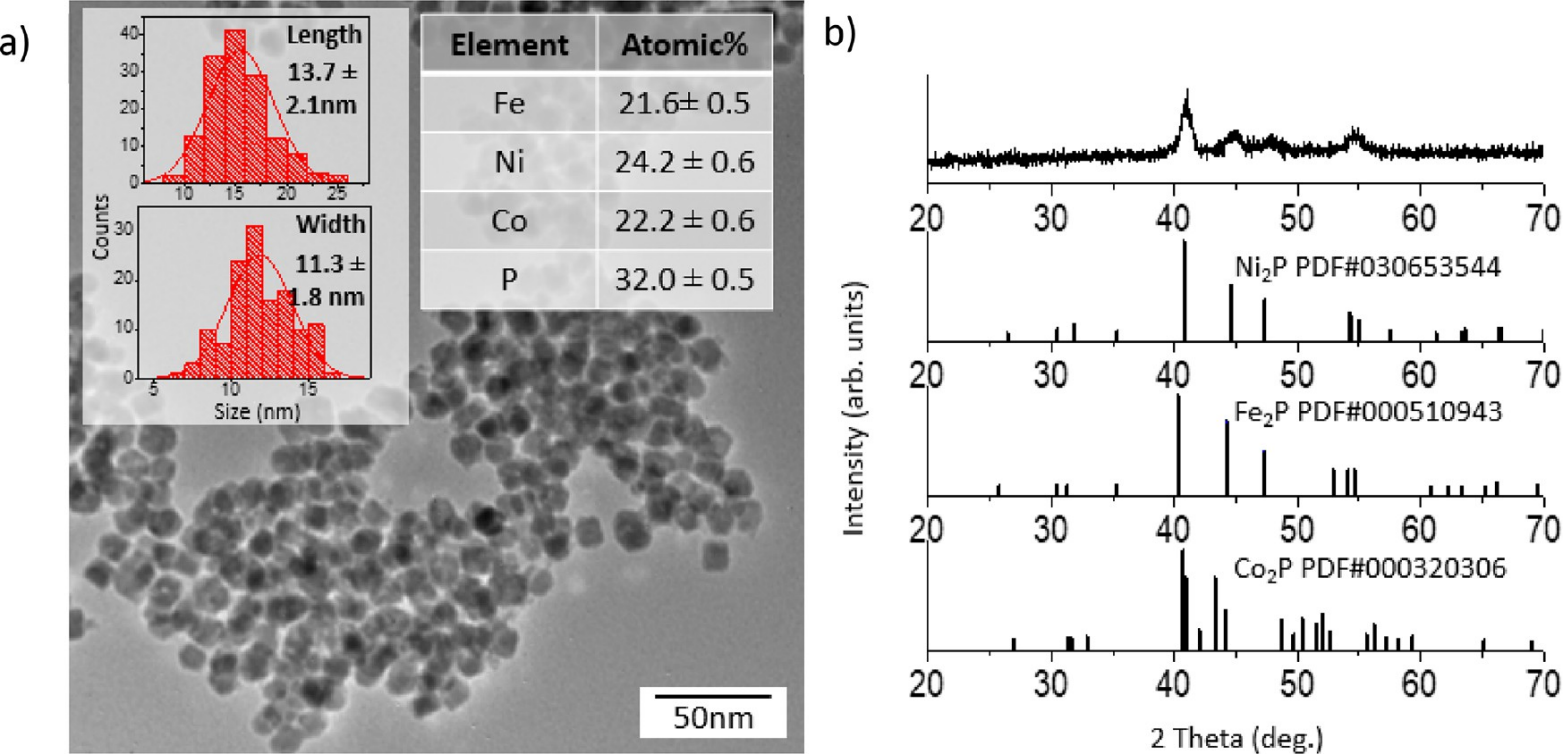
(a) TEM, EDS, and size histograms of trimetallic Fe_2–*x*–*y*_Ni_*x*_Co_*y*_P nanoparticles synthesized in a two-step reaction following Scheme 1, Path C, in which a bimetallic Ni–Co–P amorphous alloy was made first and Fe was added in an additional step, and (b) corresponding PXRD pattern.

**Figure 11:**
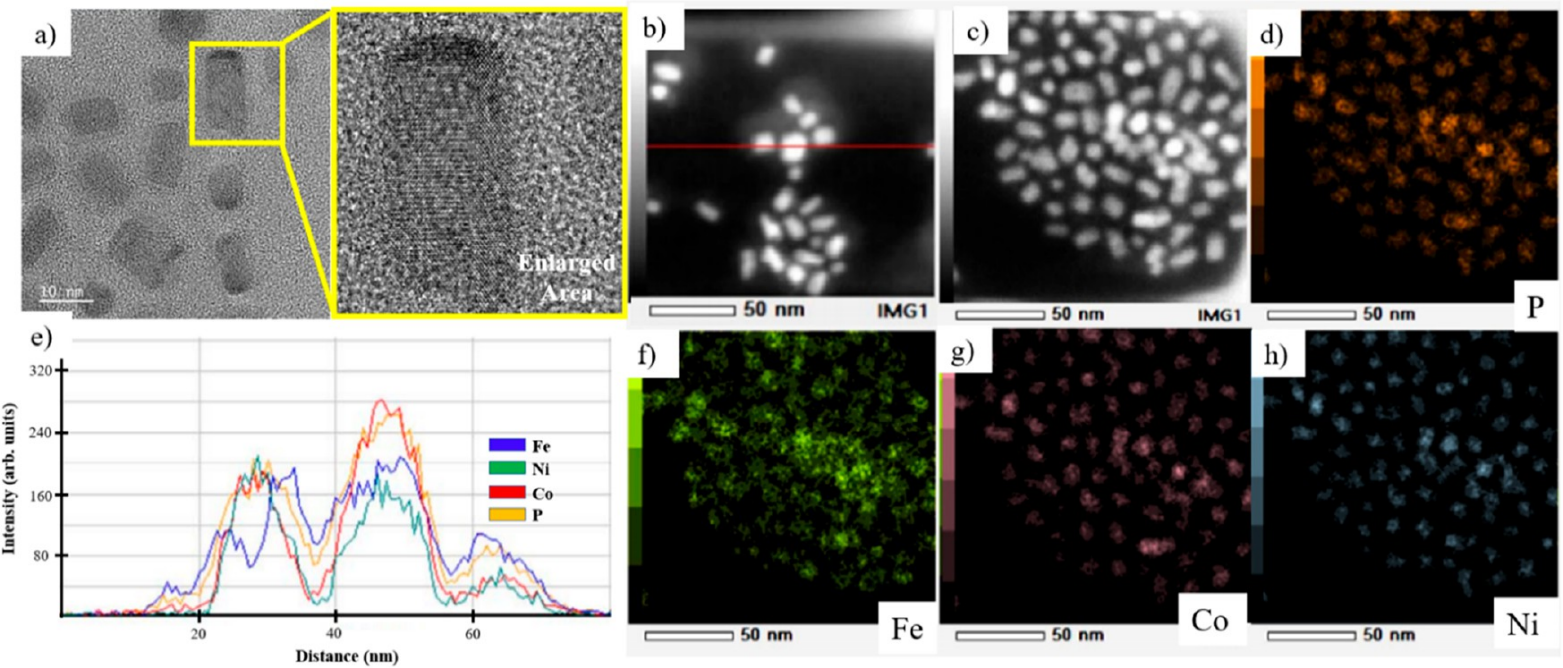
(a) HRTEM image and (b,c) HAADF images of trimetallic Fe_2–*x*–*y*_Ni_*x*_Co_*y*_P nanoparticles synthesized following Scheme 1, Path C, in which a bimetallic Ni–Co–P amorphous alloy was made first with Ni(acac)_2_ and Co(acac)_2_, and Fe(CO)_5_ was added in an additional step, crystallization *T* = 300 °C. EDS mapping data of these nanoparticles are shown for (d) P, (f) Fe, (g) Co, and (h) Ni. (e) EDS line scan across the red line following the particles imaged in panel b.

Turning to the Scheme 1, Path C two-step procedure (Scheme 1, Path C) in which a bimetallic metal phosphide amorphous alloy is synthesized first (from Ni(acac)_2_ and Co(acac)_2_) and Fe(CO)_5_ is injected in a second step, we observe monodisperse short rods with an average length of 13.7 ± 2.1 nm and width of 11.3 ± 1.8 nm with a nearly 1:1:1 ratio of metals and a 2:1 ratio of metal/P. The samples have low polydispersity and a uniform nanoparticle shape (Figure 10a). The PXRD pattern is free of impurity peaks and indicative of the Fe_2_P (hexagonal) or closely related Co_2_P (orthorhombic) structure type (Figure 10b). The HRTEM is consistent with highly crystalline nanoparticles as evidenced by the presence of clear lattice fringes (Figure 11a). EDS mapping data indicate solid solution formation as the four elements (Fe, Ni, Co, and P) are homogeneously distributed within each rod, suggesting phase segregation is absent (Figure 11b–h).

#### Compositional Variation in Fe_2–*x*–*y*_Ni_*x*_Co_*y*_P

**Figure 12:**
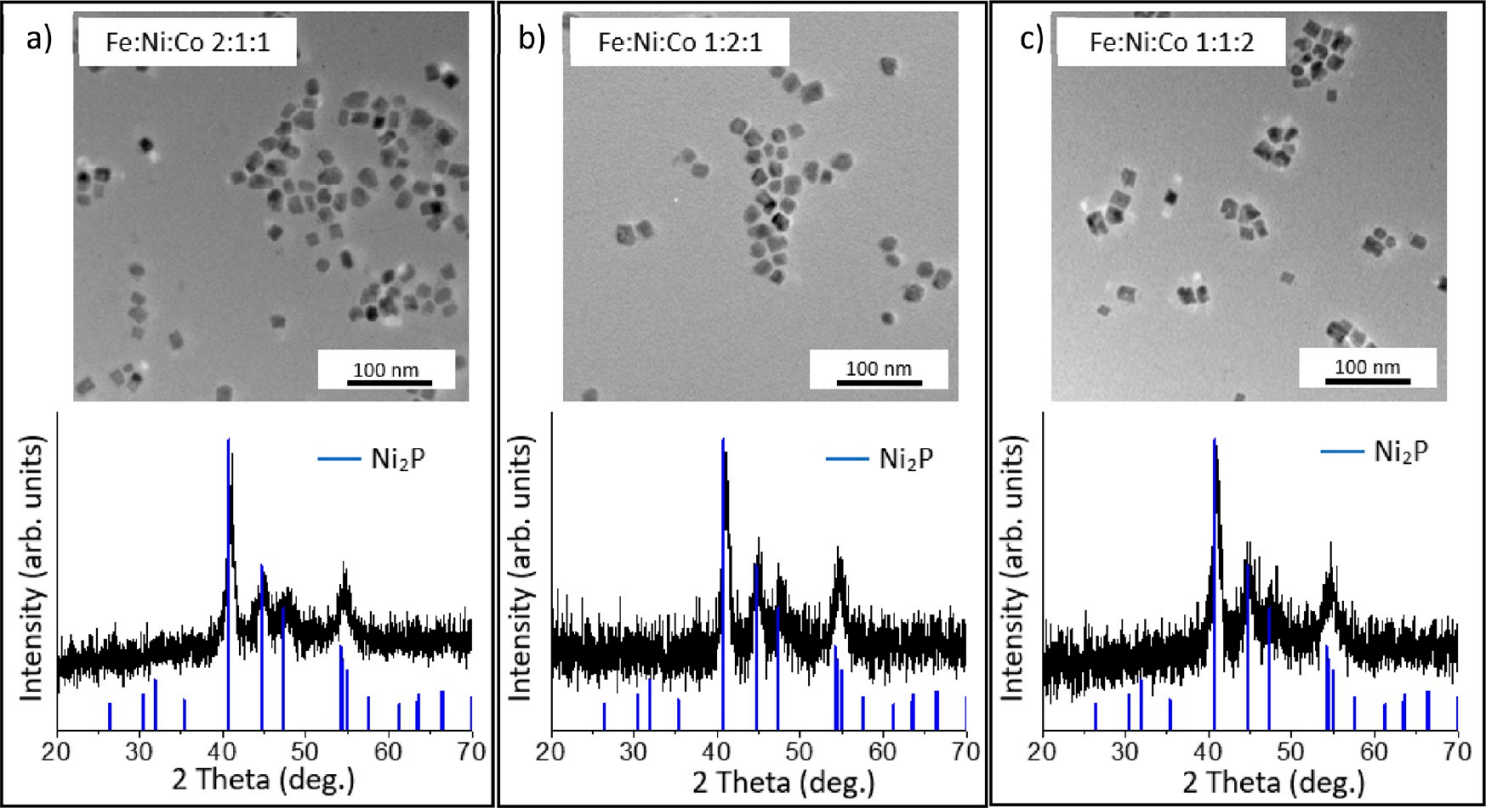
PXRD and TEM images of trimetallic Fe_2–*x*–*y*_Ni_*x*_Co_*y*_P nanoparticles synthesized in a two-step reaction following Scheme 1, Path B, in which a bimetallic Ni–Co–P amorphous alloy was made first with Ni(acac)_2_ and Co(acac)_2_, and Fe(CO)_5_ was added in an additional step, crystallization *T* = 300 °C. Targeted Fe/Ni/Co ratios were (a) 2:1:1, (b) 1:2:1, and (c) 1:1:2. Patterns matched with Ni_2_P (PDF 030653544) for comparison.

Other compositions of the trimetallic phase were pursued using the same strategy, but altering the relative ratios of the different metal precursors. In these studies, in order to evaluate the effect that modifying the initial precursor amounts had on the final composition, we employed Fe/Ni/Co ratios of 2:1:1, 1:2:1, and 1:1:2. The TEM images and PXRD patterns are shown in Figure 12, and the size histograms are presented in Figure S7. For all three targeted phases, the nanoparticles retain the short rod morphology with an aspect ratio of 1.3, and the PXRD patterns are consistent with M_2_P formation and provide no evidence of any other crystalline phases. When altering the initial Ni or Co precursor amount, the system is well behaved with target Fe/Co/Ni ratios of 1:2:1 and 1:1:2, matching closely with observed ratios of 1.1:2.1:1.0 and 1.2:1.0:1.9, respectively (Table 8). However, when the Fe/Co/Ni ratio was adjusted to 2:1:1, the observed ratio in the final nanoparticles was 1.3:1.0:1.0; that is, Fe incorporation is limited.

**Table 8:** EDS (atm %) of Fe, Ni, Co, and P for the Trimetallic Fe_2–*x*–*y*_Ni_*x*_Co_*y*_P Reactions Synthesized in a Two-Step Reaction Following Scheme 1, Path B, in Which a Bimetallic Ni–Co–P Amorphous Alloy Was Made First with Ni(acac)_2_ and Co(acac)_2_ and Fe(CO)_5_ Was Added in an Additional Step

figure	target ratio Fe/Ni/Co	Fe (atm %)	Ni (atm %)	Co (atm %)	P (atm %)	actual ratio Fe/Ni/Co
12a	2:1:1	25.5 ± 0.4	21.0 ± 0.5	20.3 ± 0.5	33.2 ± 0.3	1.3:1.0:1.0
12b	1:2:1	17.7 ± 0.7	33.3 ± 0.5	15.7 ± 0.8	33.3 ± 0.5	1.1:2.1:1.0
12c	1:1:2	19.9 ± 1.2	16.1 ± 1.5	31.2 ± 0.9	32.8 ± 0.8	1.2:1.0:1.9

Recognizing that the Fe precursor is added as a separate step after Ni–Co–P amorphous alloy formation, we considered whether Fe incorporation is limited by diffusion kinetics. In such a case, we would expect that increasing the driving force (Fe precursor concentration) and annealing time might lead to better uptake. Targeting a Fe/Ni/Co ratio of 4:1:1 following Scheme 1, Path C, doubling the post-Fe annealing time (to 2 h at 230 °C) and quadrupling the crystallization time (to 4 h at 300 °C) produced longer rod-shaped particles (aspect ratio ∼2.5) with a PXRD pattern consistent with M_2_P phase formation and an Fe/Ni/Co product ratio of 3.5:1.0:1.0 (Figure S8). Consistent with our hypothesis that Fe uptake is limited by diffusion processes, the rods are peppered with voids, suggesting relatively fast outward diffusion of Ni/Co and slow inward diffusion of Fe (the Kirkendall effect).

#### XPS Analysis of Fe_2–*x*–*y*_Ni_*x*_Co_*y*_P

In order to probe the relative oxidation states of the metals within the phosphide matrix and confirm the stoichiometry assessed by TEM/EDS, a sample of nominal composition Fe_0.94_Ni_0.54_Co_0.52_P_1.1_, as determined by TEM/EDS, was probed with XPS and the resulting spectra are presented in Figure S9. The data revealed a mixture of oxidized and reduced Fe, Co and P, whereas Ni is present only in its reduced form (Supporting Information, Table S3). The surface composition determined by XRF was Fe_0.88_Ni_0.49_Co_0.63_P_1.3_. Metal ratios are comparable to EDS analysis, but slight variation may be due in part to surface etching during the Ar sputtering process.^51,52^ These data are consistent with prior XPS data on monometallic and bimetallic phosphides of Fe, Co, and Ni.^49,53−55^

## Conclusions

The ability to rationally target solid solutions of multimetallic transition metal phosphide nanoparticles as narrow polydispersity samples with composition control is predicated on a comprehensive understanding of the relative reactivity of metal precursors under a common set of reaction conditions. Considering routine metal precursors employed in monometallic phosphides of Fe, Co, and Ni (acetylacetonate (acac) salts or metal carbonyls) plus trioctylphosphine as the P source, and common synthetic pathways involving (1) formation of an intermediate amorphous alloy at moderate temperatures (220 °C) in the presence of a reducing cosolvent (oleylamine), followed by (2) formation of crystalline M_2_P phases at 300 °C, we show that the ability to undergo phosphidation and the rate of that phosphidation are critical parameters governing solid solution formation in bimetallic and trimetallic phosphides. Notably, acac salts produce monometallic phosphide only for Ni under our standard conditions (Fe and Co produce oxides); monometallic phosphides of Fe and Co are produced when zero-valent carbonyl precursors are employed. The relative rates of phosphidation for carbonyls of Fe and Co and the acac salt of Ni reveal comparable phosphorus uptake for Ni and Co as a function of temperature, whereas P uptake by Fe becomes pronounced only at higher temperatures. Single-step formation of bimetallic Ni_2–*x*_Co_*x*_P can be readily achieved via mixing of Ni(acac)_2_ with Co_2_(CO)_8_ but also (unexpectedly) from Ni(acac)_2_ with Co(acac)_2_, with the latter reflecting facilitation of Co reduction when Ni is present. The limited phosphidation kinetics for Fe(CO)_5_ dictate two-step reactions for bimetallic phosphides Fe_2–*x*_M_*x*_P (M = Ni, Co) either by initial phosphidation of M followed by Fe introduction or by Fe–M amorphous alloy formation followed by phosphorus introduction. Finally, this knowledge was employed to target stoichiometric trimetallic phosphide nanoparticles, Fe_2–*x*–*y*_Ni_*x*_Co_*y*_P as solid solutions with narrow polydispersities from the reaction of Fe(CO)_5_ with prealloyed Ni–Co–P. These data form the basis of a “library” of reactivity data that can be used to streamline syntheses of increasingly complex colloidal phosphide phases.

## Supporting Information Available

TEM and PXRD of two step reactions in which phosphorus was added in a second step for Co(acac)_2_ and Fe(acac)_3_; TEM, PXRD, and EDS of the two step reactions following Scheme 1, Path B, for the bimetallic combinations Co(acac)_2_/Fe(acac)_3_, Ni(acac)_2_/Fe(acac)_3_, and Co(acac)_2_/Fe(CO)_5_; PXRD of aliquots retrieved from the monometallic relative rate of phosphidation study with Ni(acac)_2_; HRTEM, PXRD, and EDS maps of trimetallic TMP materials isolated from a one-step reaction using Fe(CO)_5_, Ni(acac)_2_, and Co(acac)_2_; TEM, PXRD, and EDS of alternative pathways to synthesize trimetallic Fe_2–*x*–*y*_Ni_*x*_Co_*y*_P nanoparticles; histograms of particle sizes corresponding to Figure 12; TEM, PXRD, and EDS of the 4:1:1 Fe/Ni/Co reaction employing Scheme 1, Path C; XPS analysis of Fe_0.94_Ni_0.54_Co_0.52_P_1.1_ (PDF)
