# Supplementary material for: Homogeneous Nanoparticles of Multimetallic Phosphides via Precursor Tuning: Ternary and Quaternary M2P Phases (M = Fe, Co, Ni)
Source: ACS Nanosci Au. 2022 Aug 9;2(6):503–19. doi: 10.1021/acsnanoscienceau.2c00025 (PMC9782794; doi:10.1021/acsnanoscienceau.2c00025)
Supplement: Supplementary file 1 — ng2c00025_si_001.pdf [file ng2c00025_si_001.pdf]

**SUPPORTING INFORMATION**

# Homogeneous Nanoparticles of Multimetallic Phosphides via Precursor Tuning: Ternary and Quaternary $M_2P$ phases (M = Fe, Co, Ni)

Tepora Su'a[1], Mikaylah N. Poli[1], and Stephanie L. Brock[1,*]

[1]Department of Chemistry, Wayne State University, Detroit, MI 48202 USA
*EMAIL: sbrock@chem.wayne.edu

**S1. Targeting monometallic phosphides of Fe and Co by decoupling reduction and phosphidation steps:** In one pot reactions, OAm, serving as a reducing agent, and TOP, serving as reducing agent/phosphorus source, are combined at the outset of the reaction. We hypothesized that having both of these strongly binding ligands at the outset of the reaction may encourage a competition for metal active sites, preventing the proper reduction of these di- and tri-valent metal acac salts. To test this hypothesis, we employed a two-step reaction pathway in which the metal precursor was combined with OAm and ODE initially and maintained at 230°C for 1 h before TOP was added in second step and the temperature was increased to 300°C. This was done to isolate the reduction and phosphidation steps in order to promote the homogeneous formation of the desired $M_2P$ phase. With this method, for the monometallic phases we observed that for $Co(acac)_2$, formation of $Co_2P$ was possible at 300°C through a $Co_xO_yP_z$ intermediate (**Figure S1**). However, for $Fe(acac)_3$, $Fe_3O_4$ remained the majority phase at 230°C and 300°C. Only when the temperature was raised to 330°C did a mixture of FeP and $Fe_2P$ particles become apparent via XRD and TEM analysis (**Figure S2**). This suggests that in this solvent set up and reaction conditions, oxides do not limit reactivity and formation of $M_2P$ particles may be attained with $Co(acac)_2$ and $Fe(acac)_3$ precursors, but varied synthetic parameters such as two-step procedures and elevated temperatures are necessary to chemically reduce oxide byproducts.[1] Furthermore, the most oxophilic of the three metal ions (Fe(III)) is the hardest to reduce and therefore requires higher synthesis temperatures.

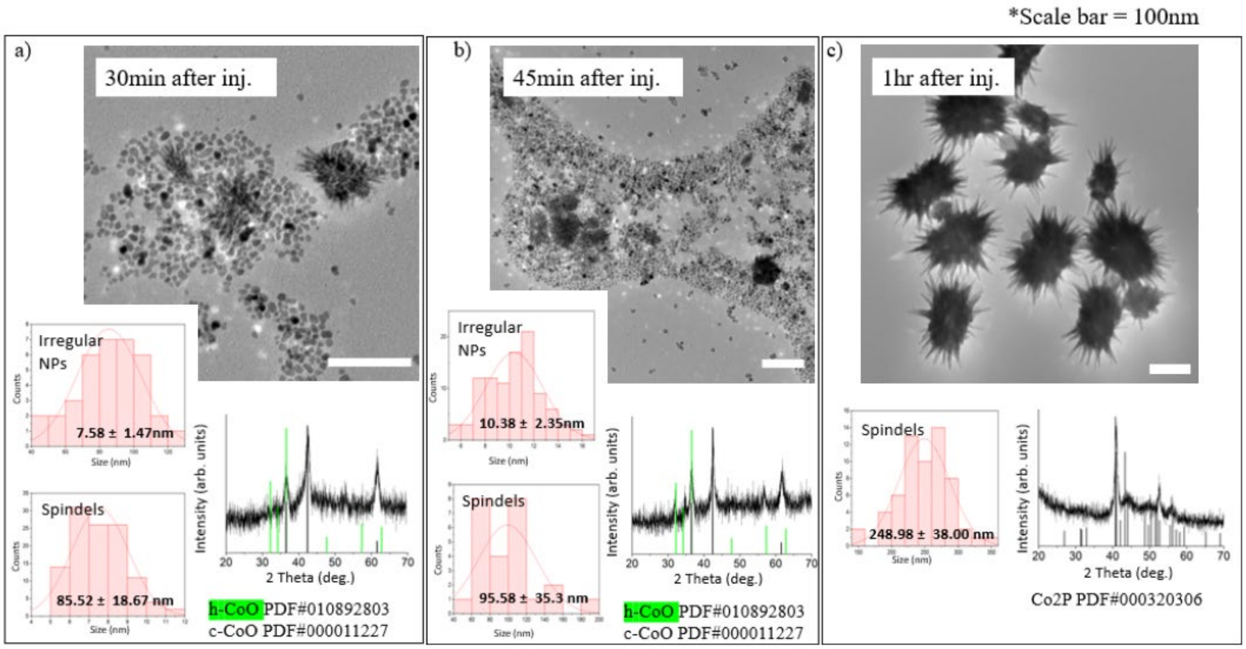


**Figure S1:** PXRD, TEM, and size distribution of isolated nanoparticles of the two step reaction where Co(acac)$_2$ was reduced and TOP was added in a second step. Aliquots taken at a) 30 min, b) 45 min, and c) 1 hr after injection of TOP. The reference patterns for h-CoO (PDF#010892803), c-CoO(PDF#00011227), and $Co_2P$ (PDF#00320306) are given for comparison. Scale bars = 100nm.

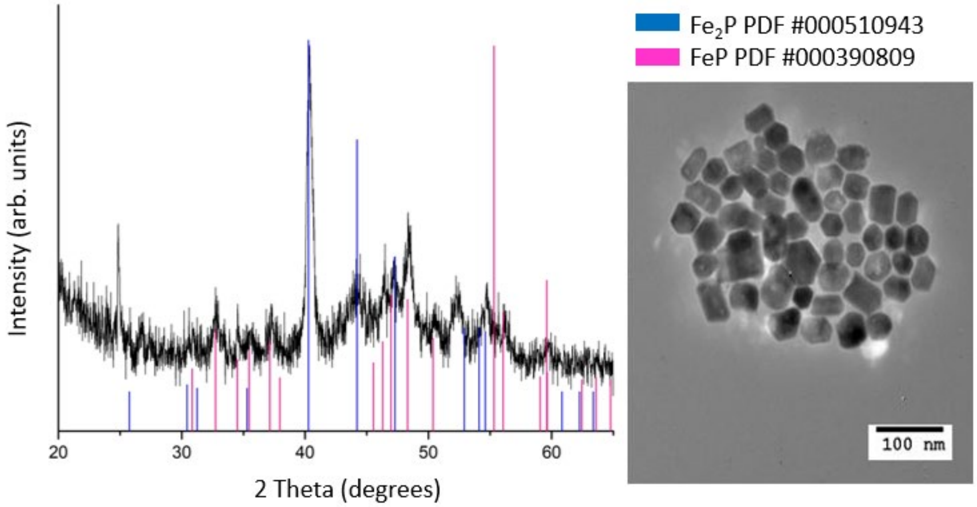


**Figure S2:** PXRD and TEM image of Fe(acac)$_3$ employed in a two step reaction at a higher temperature of 330°C. PDF patterns for $Fe_2P$ (PDF#00510943) and FeP (PDF#000390809) provided for comparison.

**S2. Targeting bimetallic phosphides from metal acetylacetonate precursors using two-step reactions (Scheme 1, Path B):** As we were able to convert the Co(acac)$_2$ precursor into the desired $Co_2P$ phase and the Fe(acac)$_3$ precursor into a mixture of FeP and $Fe_2P$ (albeit at higher temperature) using the two-step procedure in which the reduction and phosphidation steps were isolated, employing this reaction pathway to form bimetallic $Fe_{2-x}Co_xP$ seemed feasible (**Scheme 1, Path B**). However, the XRD data of the bimetallic particles isolated from this reaction pathway again matched well with the reference pattern of $Fe_3O_4$ type materials, with some shifting to lower two-theta, a possible indicator of Co incorporation into the structure (**Figure S3a, Table S1**). It should be noted that Sun and coworkers reported the conversion of CoFeO to CoFeP through a further 12hr reaction with TOP, so conversion of bimetallic oxides to phosphides is possible, but in our chosen solvent system and under the reaction conditions employed oxides were the major product.[2] The combination of Fe(acac)$_3$ and Ni(acac)$_2$ following **Scheme 1, Path B** resulted in the formation of a metal oxide/metal phosphide phase segregated product (**Figure S3b**). In this case, Fe(acac)$_3$ may be favoring the formation of iron oxide, while Ni(acac)$_2$ in the presence of OAm and TOP form the competing $Ni_2P$ byproduct, although formation of multiple bimetallic products (phosphide and oxide) is also possible. This is supported by the significant phosphidation detected by EDS for these reactions (**Table S1**).

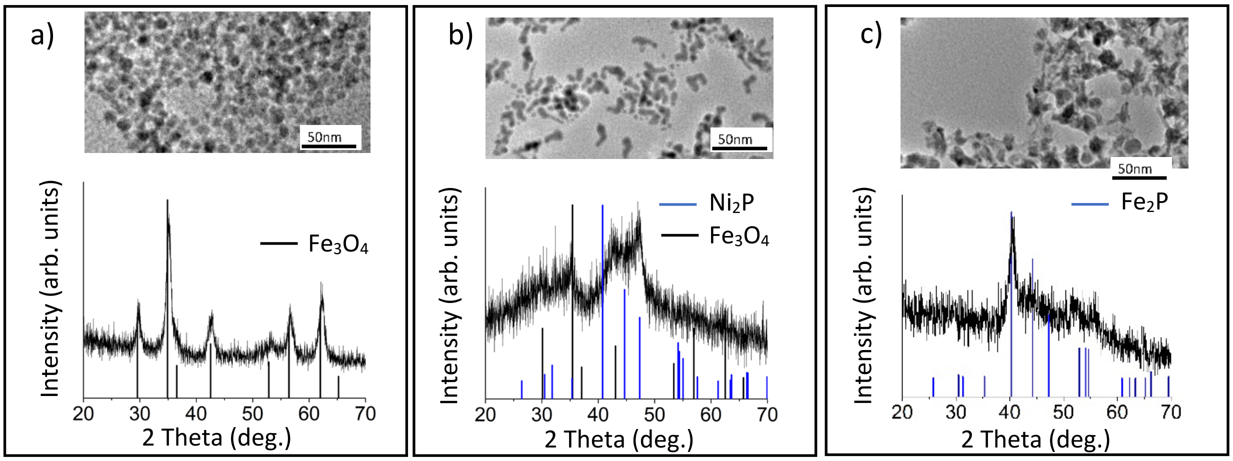


**Figure S3.** TEM and PXRD of nanoparticles isolated from two step reactions, **Scheme 1 Path B**, of a) $Co(acac)_2$ and $Fe(acac)_3$, b) $Ni(acac)_2$ and $Fe(acac)_3$, c) $Fe(CO)_5$ and $Co(acac)_2$. Reference patterns for CoO (PDF#0000011227), $Ni_2P$ (PDF# 030653544), $Fe_2P$ (PDF# PDF#00510943), and $Fe_3O_4$ (PDF#010716336) are provided for comparison.

**Table S1.** EDS atm % of Fe, Co, Ni, and P for the reactions shown in **Figure S3**.

| Fig. | Rxn | Fe (atm%) | Ni(atm%) | Co(atm%) | P(atm%) |
|---|---|---|---|---|---|
| S3a | Path B: Co-Fe + P | 53.3±0.4 | --- | 44.7±0.5 | 2.02±1.0 |
| S3b | Path B: Ni-Fe + P | 44.5±0.4 | 29.2±0.3 | --- | 26.3±0.4 |
| S3c | Path B: $Co(acac)_2$ + $Fe(CO)_5$ | 16.8±2.1 | --- | 45.2±0.6 | 38.0±0.6 |

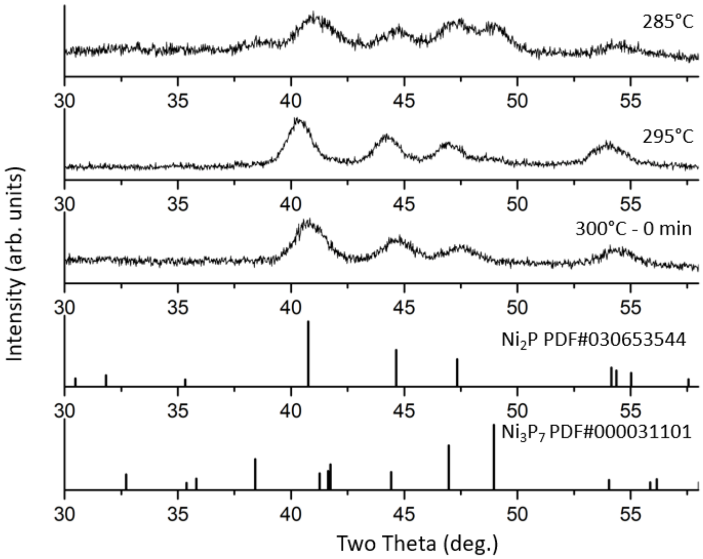


**Figure S4.** PXRD patterns of aliquots isolated when using $Ni(acac)_2$ in a one-step reaction at 285°C, 295°C, and 300°C – 0 min. References patterns for $Ni_2P$ (PDF#030653544) and $Ni_3P_7$ (PDF#000031101) provided for comparison.

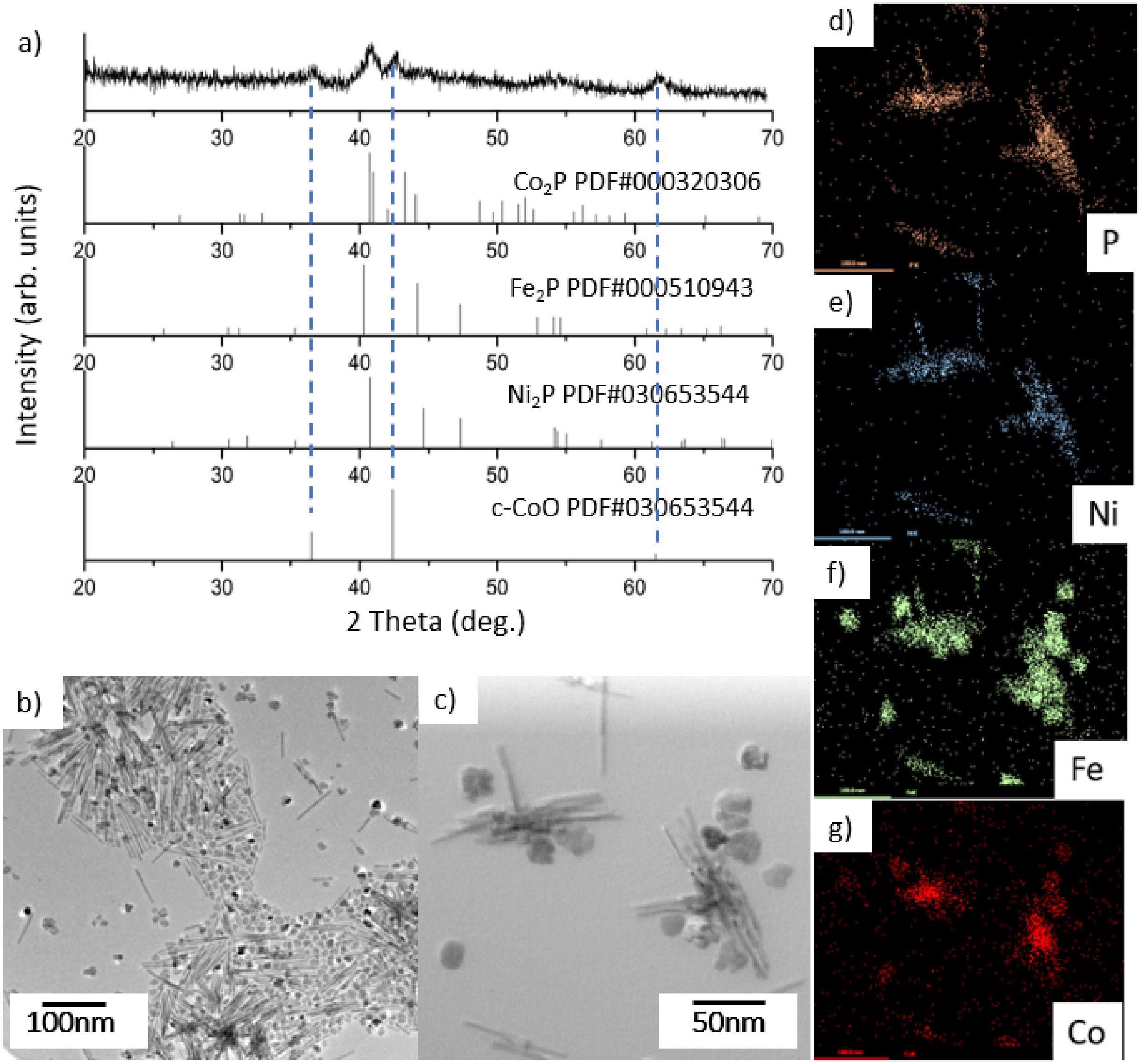


**Figure S5.** (a) PXRD (b) TEM (c) HRTEM (d) P EDS map (e) Ni EDS map (f) Fe EDS map (g) Co EDS map of nanoparticles isolated at 300°C using $Fe(CO)_5$, $Ni(acac)_2$, and $Co(acac)_2$ in a one-step reaction.

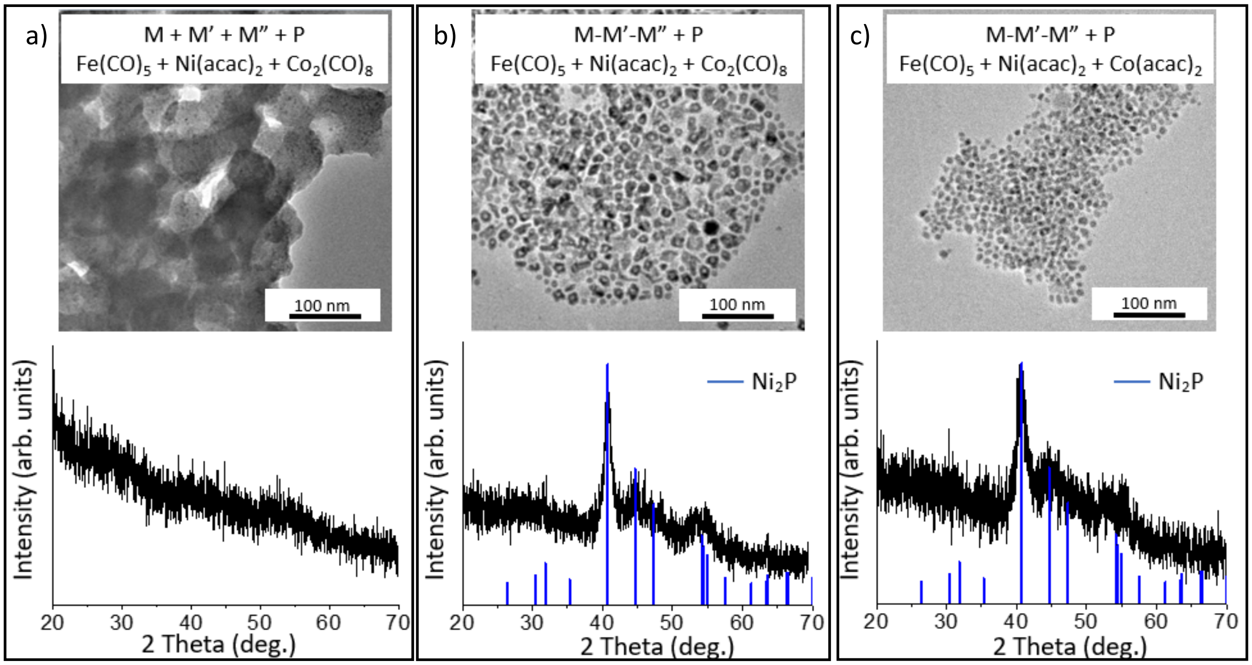


**Figure S6.** PXRD and TEM of (a) a one-step reaction with $Fe(CO)_5$, $Ni(acac)_2$, and $Co_2(CO)_8$ at 300°C (b) a two-step rection where Fe-Ni-Co amorphous particles were synthesized with $Fe(CO)_5$, $Ni(acac)_2$, and $Co_2(CO)_8$ in the first step at 230°C, and TOP is added in a subsequent step, crystallization T=300°C. (c) a two-step rection where Fe-Ni-Co amorphous particles were synthesized with $Fe(CO)_5$, $Ni(acac)_2$, and $Co(acac)_2$ in the first step at 230°C, and TOP is added in a subsequent step, crystallization T=300°C. A reference pattern of $Ni_2P$ (PDF: 030653544) is provided for comparison

**Table S2.** EDS atm % of Fe, Ni, Co, and P for the trimetallic $Fe_{2-x-y}Ni_xCo_yP$ reactions synthesized via Path A with $Co_2(CO)_8$ and Path B with $Co_2(CO)_8$ or $Co(acac)_2$.

| Fig. | Rxn | Fe (atm%) | Ni (atm%) | Co (atm%) | P (atm%) |
|---|---|---|---|---|---|
| S6a | Path A: $Fe(CO)_5 + Ni(acac)_2 + Co_2(CO)_8$ | 25.7±0.5 | 11.6±0.8 | 14.4±0.7 | 48.3±0.3 |
| S6b | Path B: $[Fe(CO)_5 + Ni(acac)_2 + Co_2(CO)_8]$ +P | 29.1±1.3 | 12.5±2.5 | 20.3±1.8 | 38.1±1.1 |
| S6c | Path B: $[Fe(CO)_5 + Ni(acac)_2 + Co(acac)_2]$ +P | 34.2±0.5 | 16.1±1.2 | 18.5±0.8 | 37.3±0.5 |

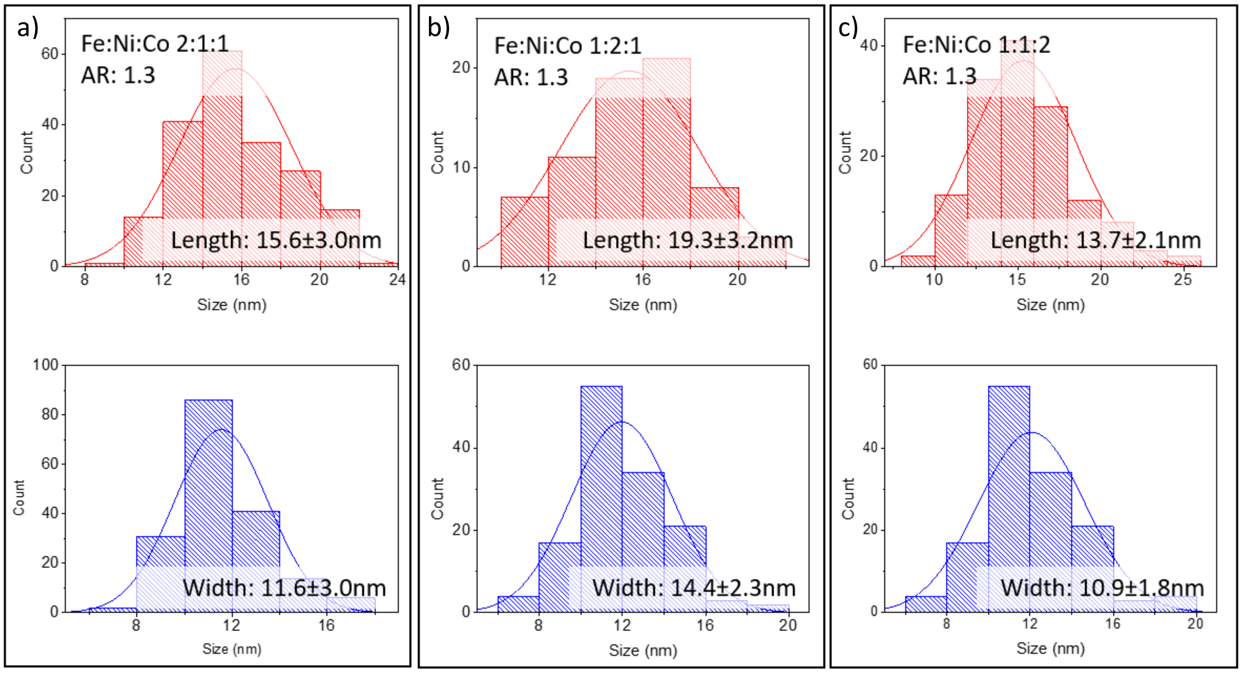


**Figure S7.** Size histograms of trimetallic $Fe_{2-x-y}Ni_xCo_yP$ nanoparticles synthesized in a two-step reaction following Scheme 1, Path B in which a bimetallic Ni-Co-P amorphous phase was made first with $Ni(acac)_2$ and $Co(acac)_2$, and $Fe(CO)_5$ was added in an additional step, crystallization T=300°C. Targeted Fe:Ni:Co ratios were a) 2:1:1 b) 1:2:1 and c) 1:1:2.

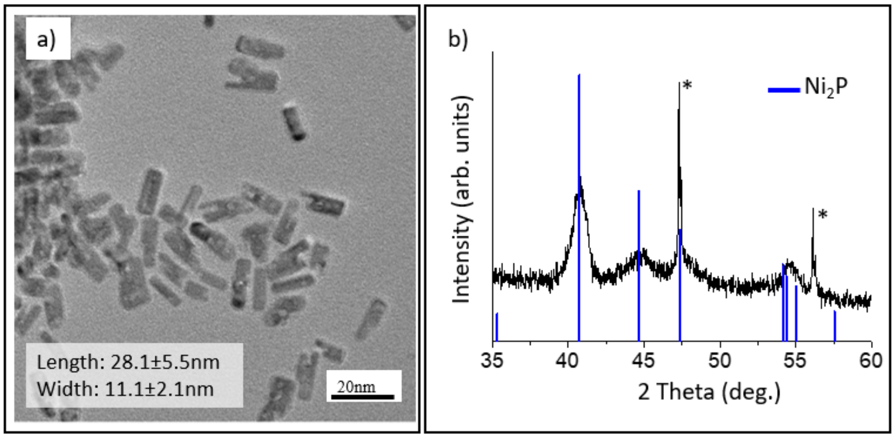


c)

| Element | Atm% |
|---|---|
| Fe | 41.4 ± 0.6 |
| Ni | 12.2 ± 1.2 |
| Co | 12.4 ± 1.2 |
| P | 34.1 ± 0.6 |

**Figure S8.** (a) TEM image (b) PXRD pattern (c) EDS analysis of trimetallic $Fe_{2-x-y}Ni_xCo_yP$ particles synthesized in a two-step reaction following Scheme 1, Path C, targeting a 4:1:1, Fe:Ni:Co initial ratio and combining a post injection annealing time of 2h and reaction time at 300°C of 4h. Asterix indicate lines for the silicon reference.

**XPS Analysis of $Fe_{0.94}Ni_{0.54}Co_{0.52}P_{1.1}$ (stoichiometry based on TEM-EDS analysis)**

XPS spectra of Fe, Ni, Co, and P are shown in Figure S9 and the binding energies and relative contributions of oxidation states and atoms are tabulated in Table S3. XPS spectra of Fe showed two types of peaks assigned to reduced $Fe^0$ (706.9 eV) and oxidized $Fe^{2+}$ (709.7 eV) and $Fe^{3+}$ (711.93eV), with the two latter ascribed to surface oxidation.[3, 4] The Ni spectra revealed a reduced Ni (870.1 eV) species consistent with reported binding energies for zerovalent Ni. Two spin-orbit doublets at 854.8 eV and 872.1 eV can be assigned to $Ni^{2+}$ (Ni $2p_{3/2}$ and Ni $2p_{1/2}$), with two satellite peaks at 860.6 eV and 877.9 eV.[5-7] The additional peak at 856.9 eV is most closely associated with $Ni^{3+}$.[8] The Co spectra showed a peak at 778.3 eV, which is consistent with the binding energy of Co in the bimetallic phosphide structure, while the 780.9 eV and 796.2 eV correspond to $Co^{3+}$ species resulting from surface oxidation.[9-11] The peaks at 778.3 and 793.1 can be assigned to $Co^{2+}$ with two satellite peaks at 797.3 eV and 802.3 eV.[9-11] XPS spectra of P showed two peaks, a resolved doublet corresponding to phosphide (P $2p_{3/2}$, 129.4 eV and P $2p_{1/2}$, 130.5 eV) and the peak centered at 134.5 eV assigned to the presence of phosphate or phosphite.[12, 13] These XPS analyses are aligned well with typical binding energies observed in $M_2P$ structure types and consistent with the formation of "$M_2P$" nanoparticles with surface stoichiometry $Fe_{0.88}Ni_{0.49}Co_{0.63}P_{1.2}$.[3, 14-16]

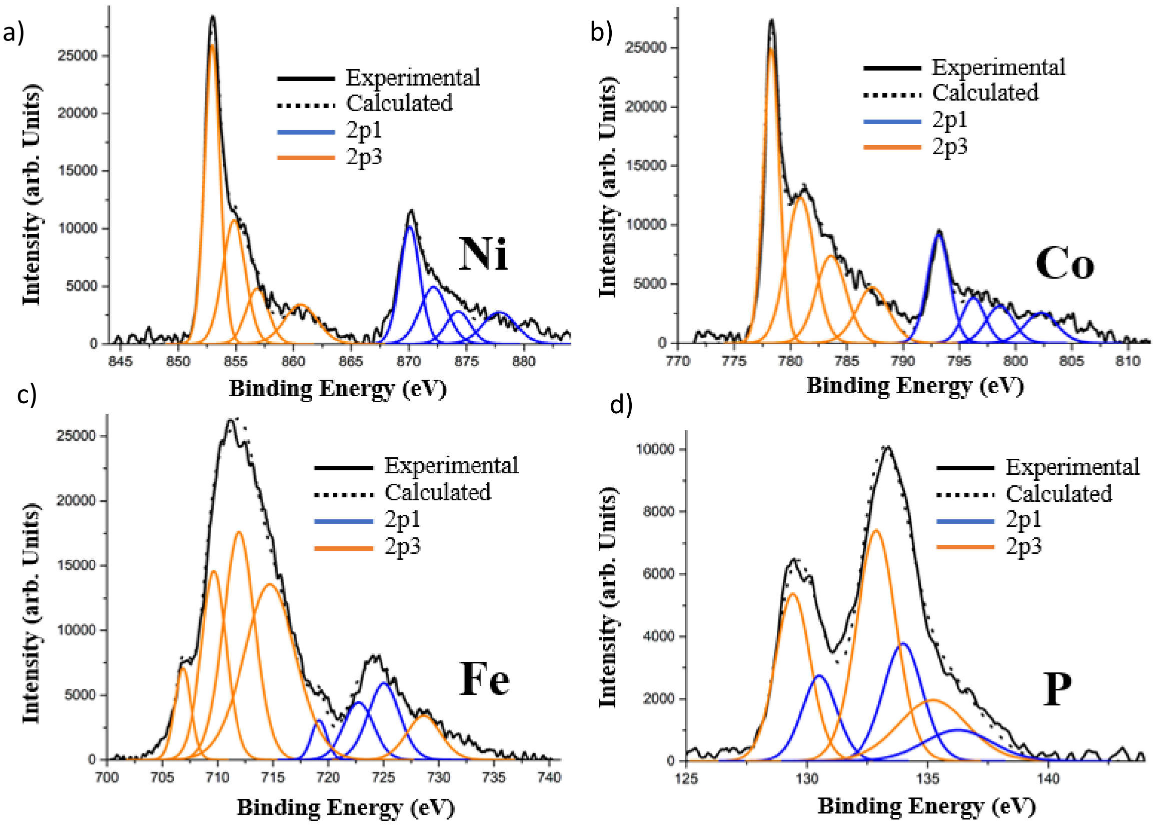


**Figure S9.** XPS fits for a) Ni, b) Co, c) Fe, and d) P of trimetallic $Fe_{0.94}Ni_{0.54}Co_{0.52}P_{1.1}$ (stoichiometry based on TEM-EDS analysis) nanoparticles synthesized in a two-step reaction following Scheme 1, Path C in which a bimetallic Ni-Co-P amorphous phase was made first and Fe was added in an additional step, targeting a 1:1:1 metal ratio. The surface stoichiometry, as assessed by XPS, $Fe_{0.88}Ni_{0.49}Co_{0.63}P_{1.2}$, is similar to the stoichiometry determined by TEM-EDS.

**Table S3.** XPS binding energies and relative oxidation state and atomic contributions for $Fe_{0.94}Ni_{0.54}Co_{0.52}P_{1.1}$ (stoichiometry based on TEM-EDS analysis). The surface stoichiometry based on XPS analysis is $Fe_{0.88}Ni_{0.49}Co_{0.63}P_{1.2}$.

| Element | Peak BE | Oxidation State | Contribution % | Overall atm% Contribution | Ref |
|---|---|---|---|---|---|
| P2p | | | | | |
| $P2p_{3/2}$ | 135.2 | $P^{5+}$ | 69.4 | 40.8 | 12,13 |
| | 132.9 | | | | |
| $P2p_{1/2}$ | 136.3 | | | | |
| | 133.9 | | | | |
| $P2p_{3/2}$ | 129.4 | $P^0$ | 30.6 | | 17,13 |
| $P2p_{1/2}$ | 130.5 | | | | |
| Fe2p | | | | | |
| $Fe2p_{3/2}$ | 711.9 | $Fe^{3+}$ | 33.5 | 26.0 | 18 |
| $Fe2p_{1/2}$ | 725.0 | | | | |
| $Fe2p_{3/2}$ | 709.7 | $Fe^{2+}$ | 59.2 | | 17, 18 |
| | 714.7 | $Fe^{2+}$ satellite | | | |
| $Fe2p_{1/2}$ | 722.8 | $Fe^{2+}$ | | | |
| | 728.6 | $Fe^{2+}$ satellite | | | |
| $Fe2p_{3/2}$ | 706.9 | $Fe^0$ | 7.3 | | 3, 4 |
| $Fe2p_{1/2}$ | 719.2 | | | | |
| Ni2p | | | | | |
| $Ni2p_{3/2}$ | 856.9 | $Ni^{3+}$ | 16.4 | 14.4 | 19 |
| $Ni2p_{1/2}$ | 874.3 | | | | |
| $Ni2p_{3/2}$ | 854.8 | $Ni^{2+}$ | 39.4 | | 5,8 |
| | 860.6 | $Ni^{2+}$ satellite | | | |
| $Ni2p_{1/2}$ | 872.1 | $Ni^{2+}$ | | | |
| | 877.9 | $Ni^{2+}$ satellite | | | |
| $Ni2p_{3/2}$ | 852.9 | $Ni^0$ | 44.2 | | 17,19 |
| $Ni2p_{1/2}$ | 870.1 | | | | |
| Co2p | | | | | |
| $Co2p_{3/2}$ | 783.6 | $Co_3O_4$ | 19.5 | 18.8 | 9, 10 |
| $Co2p_{1/2}$ | 798.6 | | | | |
| $Co2p_{3/2}$ | 780.9 | $Co^{3+}$ | 27.4 | | 9, 10 |
| $Co2p_{1/2}$ | 796.3 | | | | |
| $Co2p_{3/2}$ | 778.3 | $Co^{2+}$ | 53.1 | | 9, 10 |
| | 787.3 | $Co^{2+}$ satellite | | | |
| $Co2p_{1/2}$ | 793.1 | $Co^{2+}$ | | | |
| | 802.3 | $Co^{2+}$ satellite | | | |

References

1. Muthuswamy, E.; Brock, S. L., Oxidation Does Not (Always) Kill Reactivity of Transition Metals: Solution-Phase Conversion of Nanoscale Transition Metal Oxides to Phosphides and Sulfides. *J. Am. Chem. Soc.* **2010,** *132*, 15849-15851.

2. Mendoza-Garcia, A.; Zhu, H.; Yu, Y.; Li, Q.; Zhou, L.; Su, D.; Kramer, M. J.; Sun, S., Controlled Anisotropic Growth of Co-Fe-P from Co-Fe-O Nanoparticles. *Angew. Chem. Int. Ed.* **2015,** *54*, 9642-9645.
3. Blanchard, P. E. R.; Grosvenor, A. P.; Cavell, R. G.; Mar, A., X-ray Photoelectron and Absorption Spectroscopy of Metal-Rich Phosphides $M_2P$ and $M_3P$ (M = Cr−Ni). *Chem. Mater.* **2008,** *20*, 7081-7088.
4. Xiong, D.; Wang, X.; Li, W.; Liu, L., Facile synthesis of iron phosphide nanorods for efficient and durable electrochemical oxygen evolution. *Chem. Commun.* **2016,** *52*, 8711-8714.
5. Kim, K. S.; Winograd, N., X-ray photoelectron spectroscopic studies of nickel-oxygen surfaces using oxygen and argon ion-bombardment. *Surf. Sci.***1974,** *43*, 625-643.
6. Sawhill, S. J.; Phillips, D. C.; Bussell, M. E., Thiophene Hydrodesulfurization over Supported Nickel Phosphide Catalysts. *J. Catal.* **2003,** *215*, 343-352.
7. Moreau, L. M.; Ha, D.-H.; Zhang, H.; Hovden, R.; Muller, D. A.; Robinson, R. D., Defining Crystalline/Amorphous Phases of Nanoparticles through X-ray Absorption Spectroscopy and X-ray Diffraction: The Case of Nickel Phosphide. *Chem. Mater.* **2013,** *25*, 2394-2403.
8. Lian, K.; Thorpe, S. J.; Kirk, D. W., Electrochemical and surface characterization of electrocatalytically active amorphous NiCo alloys. *Electrochim. Acta* **1992,** *37*, 2029-2041.
9. McIntyre, N. S.; Cook, M. G., X-ray photoelectron studies on some oxides and hydroxides of cobalt, nickel, and copper. *Anal. Chem.* **1975,** *47*, 2208-2213.
10. Cabrera-German, D.; Gomez-Sosa, G.; Herrera-Gomez, A., Accurate peak fitting and subsequent quantitative composition analysis of the spectrum of Co 2p obtained with Al Kα radiation: I: cobalt spinel. *Surf. Interface Analy.* **2016,** *48*, 252-256.
11. Guo, X.; Qian, C.; Wan, X.; Zhang, W.; Zhu, H.; Zhang, J.; Yang, H.; Lin, S.; Kong, Q.; Fan, T., Facile in situ fabrication of biomorphic $Co_2P$-$Co_3O_4$/rGO/C as an efficient electrocatalyst for the oxygen reduction reaction. *Nanoscale* **2020,** *12*, 4374-4382.
12. Liu, Q.; Tian, J.; Cui, W.; Jiang, P.; Cheng, N.; Asiri, A. M.; Sun, X., Carbon Nanotubes Decorated with CoP Nanocrystals: A Highly Active Non-Noble-Metal Nanohybrid Electrocatalyst for Hydrogen Evolution. *Angew. Chem. Int. Ed.* **2014,** *53*, 6710-6714.
13. Pu, Z.; Xue, Y.; Li, W.; Amiinu, I. S.; Mu, S., Efficient water splitting catalyzed by flexible NiP2 nanosheet array electrodes under both neutral and alkaline solutions. *New J. Chem.* **2017,** *41*, 2154-2159.
14. Blanchard, P. E. R.; Grosvenor, A. P.; Cavell, R. G.; Mar, A., Effects of metal substitution in transition-metal phosphides $(Ni_{1-x}M'_x)2P$ (M′ = Cr, Fe, Co) studied by X-ray photoelectron and absorption spectroscopy. *J. Mater. Chem.* **2009,** *19*, 6015-6022.
15. Li, D.; Arachchige, M. P.; Kulikowski, B.; Lawes, G.; Seda, T.; Brock, S. L., Control of Composition and Size in Discrete $Co_xFe_{2-x}P$ Nanoparticles: Consequences for Magnetic Properties. *Chem. Mater.* **2016,** *28*, 3920-3927.
16. Liyanage, D. R.; Danforth, S. J.; Liu, Y.; Bussell, M. E.; Brock, S. L., Simultaneous Control of Composition, Size, and Morphology in Discrete $Ni_{2-x}Co_xP$ Nanoparticles. *Chem. Mater.***2015,** *27*, 4349-4357.
17. NIST X-ray Photoelectron Spectroscopy Database, NIST Standard Reference Database Number 20, National Institute of Standards and Technology, Gaithersburg MD, 20899 (2000), doi:10.18434/T4T88K, (retrieved July 2022).
18. Mills, P.; Sullivan, J. L., A study of the core level electrons in iron and its three oxides by means of X-ray photoelectron spectroscopy. *J. Phys. D: Appl. Phys.* **1983,** *16*, 723-732.
19. Du, X.; Yang, C.; Zeng, X.; Wu, T.; Zhou, Y.; Cai, P.; Cheng, G.; Luo, W., Amorphous NiP supported on rGO for superior hydrogen generation from hydrolysis of ammonia borane. *Int. J. Hydrog. Energy* **2017,** *42*, 14181-14187.
